# Spatiotemporal clusters and dengue hotspots in the Philippines: a nationwide analysis spanning 2017–2024

**DOI:** 10.3389/fpubh.2026.1781800

**Published:** 2026-04-22

**Authors:** Kenny Oriel A. Olana, Aksara Thongprachum, Napaphat Poprom, Veerasak Punyapornwithaya

**Affiliations:** 1 Doctor of Public Health Program, Faculty of Public Health, Chiang Mai University, Chiang Mai, Thailand; 2 Department of Veterinary Paraclinical Sciences, Faculty of Veterinary Medicine, Visayas State University, Baybay City, Philippines; 3 Faculty of Public Health, Chiang Mai University, Chiang Mai, Thailand; 4 Research Center for Veterinary Biosciences and Veterinary Public Health, Faculty of Veterinary Medicine, Chiang Mai University, Chiang Mai, Thailand; 5 Veterinary Public Health and Food Safety Centre for Asia Pacific (VPHCAP), Faculty of Veterinary Medicine, Chiang Mai University, Chiang Mai, Thailand

**Keywords:** dengue, Philippines, SaTScan, spatial scan, spatiotemporal clusters, trends

## Abstract

**Background:**

Spatiotemporal epidemiology of dengue remains poorly understood in the Philippines and there is scarcity of a nationwide spatiotemporal cluster analysis. This study utilizes long-term nationwide data to identify the spatial patterns and spatiotemporal clustering of dengue incidence in the Philippines.

**Methods:**

We obtained monthly data from January 2017 to December 2024 across all provinces from the Philippine Epidemiology Bureau. The data were analyzed via spatial analysis techniques, specifically Moran’s I and local Getis-Ord Gi* to determine spatial autocorrelation and hotspots. Furthermore, Poisson and space–time permutation (STP) models with varying maximum reported cluster size (MRCS) settings were applied to identify dengue spatiotemporal clusters.

**Results:**

A total of 1,903,425 dengue cases were reported in the study period, with a high concentration of cases consistently observed in the National Capital Region (NCR). Significant positive spatial autocorrelation was observed in the study period with hotspots varying across the years. Ifugao, Kalinga, Abra, Isabela and Mountain Province are the provinces most frequently identified as hotspots. Areas within the Western Visayas region were consistently identified under the primary clusters by the spatiotemporal models signifying the impact of the 2019 epidemic in the region. Compared with the Poisson models, the STP model had identified more clusters with smaller radii.

**Conclusion:**

To our knowledge, this is the first spatiotemporal cluster analysis in the Philippines on reported dengue cases at the national scale using spatial scan statistics. The study demonstrated the application of varying MRCS which has effectively detected meaningful clusters. These findings offer health agencies and authorities in the Philippines approaches to further understand disease epidemiology, particularly in terms of spatial and spatiotemporal clustering, which consequently enables the implementation of targeted interventions and resource allocation.

## 1 Introduction

Dengue, an acute emerging viral infectious disease caused by the *flavivirus* dengue virus (DENV) predominantly transmitted by the *Aedes aegypti* mosquito, is now considered endemic in 100 countries and remains to be of global health concern (1, 2). Dengue incidence is highly heterogeneous, with outbreaks emerging as localized clusters that shift over time highlighting the need for spatiotemporal approaches to surveillance and control. Despite the goal of the United Nations Sustainable Development Goals (SDGs) to halt epidemics by 2030, the incidence of dengue continues to increase globally with more than 3.8 billion people at risk by living in endemic countries and territories (2, 3). Among all the affected regions, Southeast Asia and the Western Pacific region are some of the most severely affected regions with more than 1.8 billion people at risk (4). The Philippines, a dengue-endemic country, having the highest number of dengue cases in the region, contributes greatly to the global burden of dengue (5). The disease highly impacts the country, with an estimated adjusted annual average number of dengue cases of more than 800,000, and incurs a substantial economic burden of approximately $345 million or ($3.26 per capita) (6). Interestingly, because the country is an archipelago characterized by thousands of islands with complex climate patterns (7) and rich biodiversity (8), dengue transmission may be highly spatially heterogeneous which presents a unique challenge for control. The country implements the National Dengue Prevention and Control Program (NDPCP) which aims to reduce dengue morbidity through surveillance, outbreak response, case management and diagnosis, health promotion and advocacy, integrated vector management, and research (9), however, achieving these goals depends on timely surveillance informed by spatiotemporal analysis. At present, nationwide spatial and temporal modeling of dengue occurrence remains limited in the Philippines.

Trends of dengue research in the Philippines reveal scarcity of spatial and temporal modeling particularly of national scale. Despite being considered the most well-studied mosquito-borne disease in the country, only three modeling studies were found in the Philippines for the past 60 years on dengue research and one for spatial modeling for dengue risk maps (10, 11). In terms of the application of space–time cluster detection techniques only one study was found (12). Spatiotemporal analysis of previous studies are of city or regional scope only and the data used are now outdated (9, 13, 14). Although prior spatial studies have identified dengue hotspots in specific provinces and cities, their findings are not directly transferable to national surveillance and prone to limited external validity because dengue transmission drivers and reporting systems vary substantially across ecological and administrative contexts. Differences in spatial scale, study period, covariate selection, and analytic methods limit cross-region comparability and preclude equitable national prioritization. National dengue policy requires standardized, comparable risk estimates across all jurisdictions to guide allocation of prevention and response capacity. The limitations of previous studies limit our knowledge of the hotspots emerging from other areas of the country and creates a gap in understanding the spatiotemporal epidemiology of dengue in the Philippines. This highlights the importance of employing spatiotemporal cluster detection methods to ensure timely surveillance capturing current disease dynamics, monitor trends, and detect outbreaks as well as emerging pathogens (15).

Spatiotemporal analysis plays a significant role in disease surveillance, control, and prevention. It provides insights into the spatial and temporal dynamics of dengue transmission and vector distribution, which could help predict disease outbreaks and enable tailor-fitting of public health interventions (16, 17). Lan and Delmelle (12) systematically reviewed the techniques utilized for space–time cluster detection of infectious diseases. The most common methods employed are Kuldorff’s space–time scan statistic, global and local Moran’s I, and local Getis-Ord Gi*. In addition, these methods are frequently applied for diseases such as dengue fever (22.8%), COVID-19 (16.7%), tuberculosis (13.1%), and malaria (8.6%). Majority of these studies were from China (*n* = 76), the US (*n* = 35), or Brazil (*n* = 31). No studies from the Philippines were identified.

Methods to detect spatial autocorrelation and space–time scan statistics are widely used in epidemiological studies on dengue and were chosen to delineate hotspots that could facilitate targeted intervention implementation, logical resource allocation, and efficient control and prevention (16, 18). Recent studies have used global (16, 19) and local Moran’s *I* (4, 16, 17, 19) to determine the spatial autocorrelation of the incidence rate, to indicate clustering somewhere in the study area, and to locate locations of clusters (20). In contrast, local Getis-Ord Gi* was used to identify spatial hotspots (4, 9). To detect the space–time interaction of dengue outbreaks, Kuldorff’s space–time scan statistic is commonly used (13, 19, 21–26).

This study aimed to characterize the spatiotemporal distribution, spatial clustering, and identify where and when clusters emerged using two complementary modeling approaches across Philippine provinces from 2017 to 2024. Furthermore, we evaluated the application of varying maximum reported cluster sizes in specifying cluster locations and counts. This study facilitates the understanding of the spatiotemporal epidemiology of dengue in the Philippines. It also advances the knowledge on the application of spatial scan statistics, particularly on the use of different levels of maximum reported cluster sizes and further provides useful information for policymaking regarding proper resource allocation and targeted disease control implementation.

## 2 Methods

### 2.1 Study location

The Philippines is a Southeast Asian country situated in the western Pacific Ocean, approximately 11° 41′52″N latitude and 122° 37′18″E longitude. It is composed of approximately 7,641 islands, which are classified into three main island groups: Luzon, Visayas, and Mindanao (27). The total population is more than 115 million, with a population density of 389 persons per square kilometer (28). The country has a tropical marine climate influenced by the Northeast (December–April) and Southwest Monsoons (May–November), which define its wet and dry seasons (29). Climate zones of the country has proven to be complex with six climate zones identified (7). In the Philippines, dengue transmission occurs primarily through the urban-dwelling mosquito vector *Aedes aegypti*, while *Aedes albopictus* functions as a secondary vector predominantly found in rural environments (30, 31). The most highly urbanized cities of the Philippines are Quezon City, Pasay City, the City of Manila, Muntinlupa City, which are under the National Capital Region; and Iloilo City of the Western Visayas region (32).

### 2.2 Data

The monthly number of reported dengue cases in the Philippine provinces and chartered cities from January 2017 to December 2024 was obtained from the Philippine Department of Health, Epidemiology Bureau. A total of 1,903,425 dengue cases were reported in the study period, spanning 96 months of data. As recognized by the WHO as a dengue-endemic country, all cases under suspect, probable, and confirmed classifications are systematically reported to the bureau following the case classification from the Manual of Procedures for the Philippine Integrated Disease Surveillance and Response 2014 (following the WHO Guidelines 1997) (33, 34). A suspected case is a previously healthy individual who develops an acute febrile illness lasting 2–7 days and shows clinical features consistent with dengue. A probable case is a suspected case with supporting laboratory evidence, at minimum a complete blood count (CBC) demonstrating leukopenia, with or without thrombocytopenia, and/or a positive dengue NS1 antigen test or antibody testing (e.g., IgM ELISA). A case is considered confirmed when viral isolation by culture is positive or when polymerase chain reaction (PCR) testing detects the virus. Cases detected by the hospital Epidemiology and Surveillance Units (ESUs) are reported and validated at the provincial or city ESU via the Philippine Integrated Disease Surveillance and Response Information System (PIDSR-IS). These reports had the final validation before submission to the National Epidemiology Bureau at the regional ESU.

The estimated human population by province and by month was then derived from the population growth rates from 2015 and 2020 censuses of the Philippine Statistics Authority (28). Monthly population growth rates were then calculated and applied for population estimation via the exponential population projection formula (35). The estimation assumes a province-specific constant annual growth rate between census years. This allows growth to vary across provinces (spatial heterogeneity), while remaining constant over time within each province during the intercensal period to promote parsimony and ensure comparability across provinces and years (36). The entire population of the province was considered the population at risk.

### 2.3 Data analysis

#### 2.3.1 Descriptive statistics and mapping

Descriptive statistics on cases and incidence were calculated. The incidence per 100,000 people of each province was generated yearly from 2017 to 2024 by dividing the annual total reported dengue cases by the median estimated population of the respective year and multiplying by 100,000. Dot maps of cases across the study period were generated via the “rnaturalearthhires” and “ggplot2” packages in R software (Version 4.5.1, R Foundation for Statistical Computing, Vienna, Austria) to determine the spatial distribution of the disease in the country. Choropleth maps of incidence were then created via Philippine administrative boundary shapefiles from the National Mapping and Resource Information Authority (37) in QGIS version 3.36 Maidenhead (38). Quintiles of the incidence of the entire study period were used as categories to examine the spatial patterns across the years.

#### 2.3.2 Spatial autocorrelation and hotspot analysis

Moran’s I test was employed to determine the level and patterns of provincial spatial clustering and Getis-Ord Gi* statistic to further identify the significance and extent of these clusters (39). These techniques are variations of a cross-product statistic that can be used to assess the spatial autocorrelation of ordinal or continuous attribute data (20, 40). Both techniques generate a similarity index that is weighted by proximity. The proximity matrix (spatial weights) reflects the spatial arrangement of the observations. If spatial proximity has no impact on the risk of infection, the occurrence of infection would be expected to follow a random distribution across the study area. However, a clustered spatial pattern indicates the presence of a contagious process or a localized risk factor (20).

First, a row standardized k-nearest neighbor (kNN) spatial weights matrix was constructed to reflect the Philippines’ archipelagic setting and dengue transmission dynamics. Although administrative units may be separated by water and lack direct contiguity, dengue risk can remain spatially interdependent through routine inter-island mobility and connectivity networks (e.g., travel and trade) (41, 42). kNN provides a consistent neighborhood structure that captures cross-island interconnectedness while avoiding disconnected components common in contiguity-based weights. Sensitivity analyses of the spatial weights specification were conducted by varying the k-nearest neighbors (kNN) parameter and evaluating consistency across Global Moran’s I, local Moran’s I or Local Indicators of Spatial Association (LISA), and the local Getis–Ord Gi* statistic. Across the tested values, *k* = 5 provided the most appropriate balance between statistical stability and spatial interpretability, hence the value was used for constructing the kNN spatial weights matrix. Spatial weight matrix generation and sensitivity analyses were performed via “rgeoda,” “spdep,” “sf,” “dplyr,” “ggplot2,” and “patchwork” R packages. Detailed sensitivity results are provided in Supplementary Figures 3–7. This matrix was then applied to spatial autocorrelation and hotspot analyses.

The global spatial autocorrelation test was performed to examine the overall clustering of dengue incidence for each year. The Moran’s I statistic is expressed by the following formula (40, 43):


$$I=\frac{n}{s_{0}}\frac{\sum_{i=1}^{n}\;\sum_{j=1}^{n}\;w_{i,j}x_{i}x_{j}}{\sum_{i=1}^{n}\;x_{i}^{2}},$$

where n refers to the total number of features (i.e., provinces involved in the analysis). The terms $x_{i}$ and $x_{j}$ represent the deviations of an attribute for features i and j from their respective means, and $w_{\mathrm{ij}}$ is the spatial weights matrix between features i and j . $S_{o}\;$ is the aggregation of all spatial weights. The null hypothesis of Moran’s I test posits that dengue incidence values are spatially independent—that is, measurements at any given location have no relationship with measurements at neighboring locations, suggesting a random geographical distribution of cases (20). The Moran’s I statistic ranges from −1 to 1, where values approaching −1 indicate spatial dispersion, where neighboring units exhibit dissimilar values, values approaching 1 indicate spatial clustering, where neighboring units exhibit similar values, and values near 0 suggesting a pattern consistent with complete spatial randomness.

To detect and characterize spatial clustering patterns in dengue incidence, two complementary local spatial statistics were applied: Local Moran’s I and the Getis-Ord Gi* statistic. The Local Moran’s I technique disaggregates the global Moran’s I measure into individual local values, providing spatial autocorrelation measurements for each specific area within our study region (20). This allows visualization of the degree to which values are clustered near an observation, identifying local hotspots and cold spots (4, 44).

The local Moran’s *I* is expressed by the equation (44):


$$I_{i}=\frac{x_{i}-\bar{x}}{S_{i}^{2}}\sum_{i=1,j\neq 1}^{n}\;\omega_{i,j}(x_{j}-\bar{x}),$$

The term $x_{i}$ is the attribute feature of i , and $\bar{x}$ is the mean of variable x . The term $w_{\mathrm{ij}}$ represents the spatial weights matrix between features i and j . n represents the total number of functions, and the variance is presented as:


$$S^{2}=\frac{\sum_{j=1,j\neq i}^{n}\;{\left(x_{j}-\bar{x}\right)}^{2}}{n-1}$$

Built on the Local Moran’s *I* analysis, the study region was categorized into four distinct spatial patterns (44): (1) hotspot regions (high–high associations), representing areas of elevated dengue incidence bordered by similarly high-incidence neighboring territories; (2) cold spot regions (low–low associations), indicating areas with minimal dengue activity surrounded by territories also exhibiting low case counts; (3) spatial anomalies (high-low or low-high associations), identifying territories where dengue incidence substantially differs from surrounding areas, either as isolated high-incidence zones amid low-incidence territories or as low-incidence areas surrounded by high-incidence territories; and (4) non-associated regions, denoting areas without significant statistical spatial clustering patterns (*p* > 0.05).

To examine the stability and intensity of hotspot and cold spot clusters, a local Getis-Ord (Gi*) statistical analysis was also conducted (20, 43). The Gi* statistic compares the local mean incidence (defined as the rates for a target location and its adjacent neighborhoods) to the global mean incidence (comprising the rates across all study locations). The Gi* statistic generates a *Z* score along with the corresponding *p* value for each location, identifying significant differences between the local and global means. Locations with larger, positive statistically significant *Z* scores indicate pronounced clustering of high values (hotspots), where the spatial aggregation of elevated values is highly unlikely to result from random spatial processes. Conversely, locations with smaller, negative statistically significant *Z* scores represent concentrated clusters of low values (cold spots). This spatial autocorrelation statistic $G_{i}^{*}\;$ was developed by Getis and Ord and is expressed as (43):


$$G_{i}^{*}=\frac{\sum_{j=1}^{n}\;w_{i,j}x_{j}-\bar{X}\sum_{j=1}^{n}\;w_{i,j}}{S\sqrt{\left([n\sum_{j=1}^{n}\;w_{i,j}^{2}-{\left(\sum_{j=1}^{n}\;w_{i,j}\right)}^{2}]/n-1\right)}},$$

where $x_{i}$ is the original observation of feature j , $w_{\mathrm{ij}}$ represents the spatial weights between features i and j , and n represents the number of observations.

Dengue transmission hotspots represent critical geographic areas that disproportionately contribute to disease spread, thus warranting targeted intervention strategies. Areas with persistently low transmission rates, or coldspots, provide valuable opportunities to investigate protective factors and generate hypotheses that could inform broader prevention and control measures. Spatial outliers, characterized by areas of high transmission surrounded by low transmission zones (high-low) or isolated low transmission areas within high transmission regions (low-high), merit specialized epidemiological attention, as understanding these patterns is crucial for both reducing transmission risk and protecting vulnerable populations in adjacent areas. These analyses were performed via “rgeoda” R package with 999 permutations for randomization inference and *p* < 0.05 as significance threshold.

#### 2.3.3 Spatiotemporal models

The space–time scan statistic functions as a cluster identification technique that detects geographic and temporal domains where observed case frequencies significantly exceed the expected count under conditions of random distribution. This approach detects anomalous concentrations of cases within specific geographical areas and time intervals (20). Discrete Poisson and space–time permutation (STP) were applied to detect and examine disease clusters (45, 46). The Poisson model incorporates factors such as the number of cases, population in each unit, unit coordinates, and onset date of outbreaks. In the study, the monthly population of each province used in the Poisson model was estimated using exponential population projection as described above. In contrast, the STP model utilizes case data alongside geographic coordinates and the onset date of the epidemic (46). Using both models enabled us to (i) identify space–time clusters of elevated dengue risk relative to the population at risk using census-based provincial population estimates and (ii) assess the robustness of detected clusters to potential misspecification of population denominators and heterogeneous reporting across provinces and over time using a case-only space–time permutation analysis. Thus, clusters detected consistently by both models provide stronger evidence of a true outbreak-like signal, a concentration of cases that is localized in both space and time and unlikely to be an artifact of population denominator error or reporting heterogeneity.

Retrospective spatiotemporal analysis was employed to identify dengue hotspots through space–time scan statistical methodology, thereby determining geographical areas and time periods exhibiting significantly elevated dengue risk. Implementation of the space–time scan statistic involves a cylindrical scanning window whose circular base is positioned sequentially at each provincial centroid throughout the defined study region. The cylindrical scanning window uses its base radius to define the geographic scope of potential clusters, while the cylinder’s height establishes the period under examination. These parameters are methodically adjusted throughout the analysis to identify clustering patterns across various geographic extents and time intervals. Under the null hypothesis, the spatial and temporal distributions of cases within each province are assumed to follow a Poisson distribution with uniform risk across both space and time. The maximum spatial cluster size and temporal window were set to 50% of the total population at risk and during the study period, respectively, based on default parameters to prevent multiple testing issues (47). The data were aggregated by province and month. To report secondary clusters, a no geographical overlap criterion was used.

First, the dynamic window shifts both spatially and temporally to encompass all potential geographic locations, sizes, and periods (48). A log-likelihood ratio (LLR) for each cylindrical window was computed by contrasting the number of observed cases with expected case frequencies inside and outside the defined window parameters. To evaluate statistical significance, a Monte Carlo simulation procedure with 999 randomized iterations was implemented, testing the null hypothesis that relative risk remained uniform across regions. The cylindrical window generating the highest LLR was designated as the primary cluster, while additional windows demonstrating statistical significance were classified as secondary clusters. A significance threshold of *p* < 0.05 was employed to determine statistical validity of identified clusters. Varied maximum reported cluster size settings (5, 10, 15, 30, 50%) were applied to refine the models (49). These analyses were conducted using R software as an automated wrapper, employing the “dplyr,” “readr,” and “rsatscan” R packages, with linkage to SaTScanBatch64.exe in SaTScan version 10.1.3 (50). The identified statistically significant clusters were mapped via QGIS (38).

The R codes used for spatial autocorrelation, hotspot analysis, and sensitivity analysis are provided. In addition, example R codes for the spatiotemporal models are included for selected parameter settings, serving as a reference for the configurations applied in this study and for further parameterization and model development (Supplementary material 2).

## 3 Results

### 3.1 Descriptive analysis and spatial distribution

From January 2017 to December 2024, the Philippines recorded a high number of dengue cases, accounting for 1,903,425 cases ranging from 80,919 to 437,089 annually, with a median of 216,874 cases. The mean monthly dengue incidence averaged 17.66 ± 15.97 (mean ± standard deviation) per 100,000 people. The disease occurs throughout the year, but its seasonality is notable, with the monthly dengue incidence peaking between July and September (Supplementary Figure 1). The highest number of recorded total annual dengue cases was in 2019 (437,089), and the lowest number was in 2021, with 80,919 cases. A great resurgence has occurred in 2024 with 413, 960 cases.

**Figure 1:**
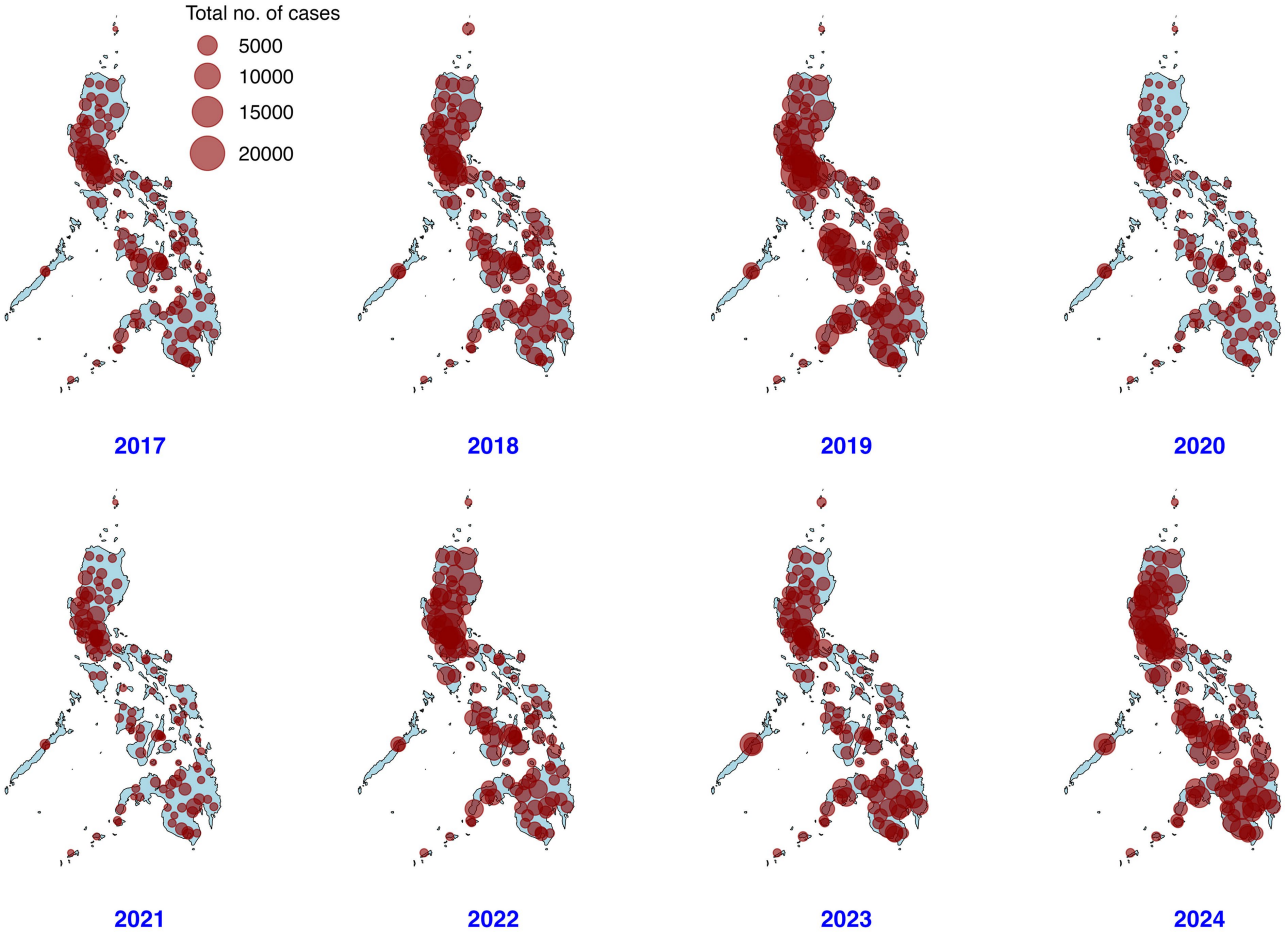
Dot map of dengue cases in the Philippines from January 2017–December 2024. The dots represent the centroids of the reporting provinces and chartered cities; their size characterizes the number of cases. The national administrative boundaries of the Philippines were generated using the “rnaturalearthhires” data package in R.

Dengue has occurred in all provinces of the country. Figure 1 presents a dot map of dengue cases by province and reporting city from 2017 to 2024. A high concentration of cases was consistently observed in the National Capital Region (NCR) throughout the study period. In 2019, most of the provinces recorded a high number of cases, with a median of 2,166 cases (interquartile range (IQR) = 1,121–4,301). The provinces with the highest number of cases in 2019 were Iloilo (23,043), Laguna (21,837), and Cavite (21,787) while in 2024, the provinces of Cebu (14,910), Cavite (14,905) and Bulacan (12,946) topped cumulative totals. The epidemic in 2019 heavily impacted the country, with more than 70% (85/121) of the epidemiology surveillance units reported more than 300 cases per 100,000 people. Considering the human population, variations in incidence per 100,000 were observed in all the provinces (Supplementary Figure 2).

### 3.2 Spatial autocorrelation and hotspots

**Figure 2:**
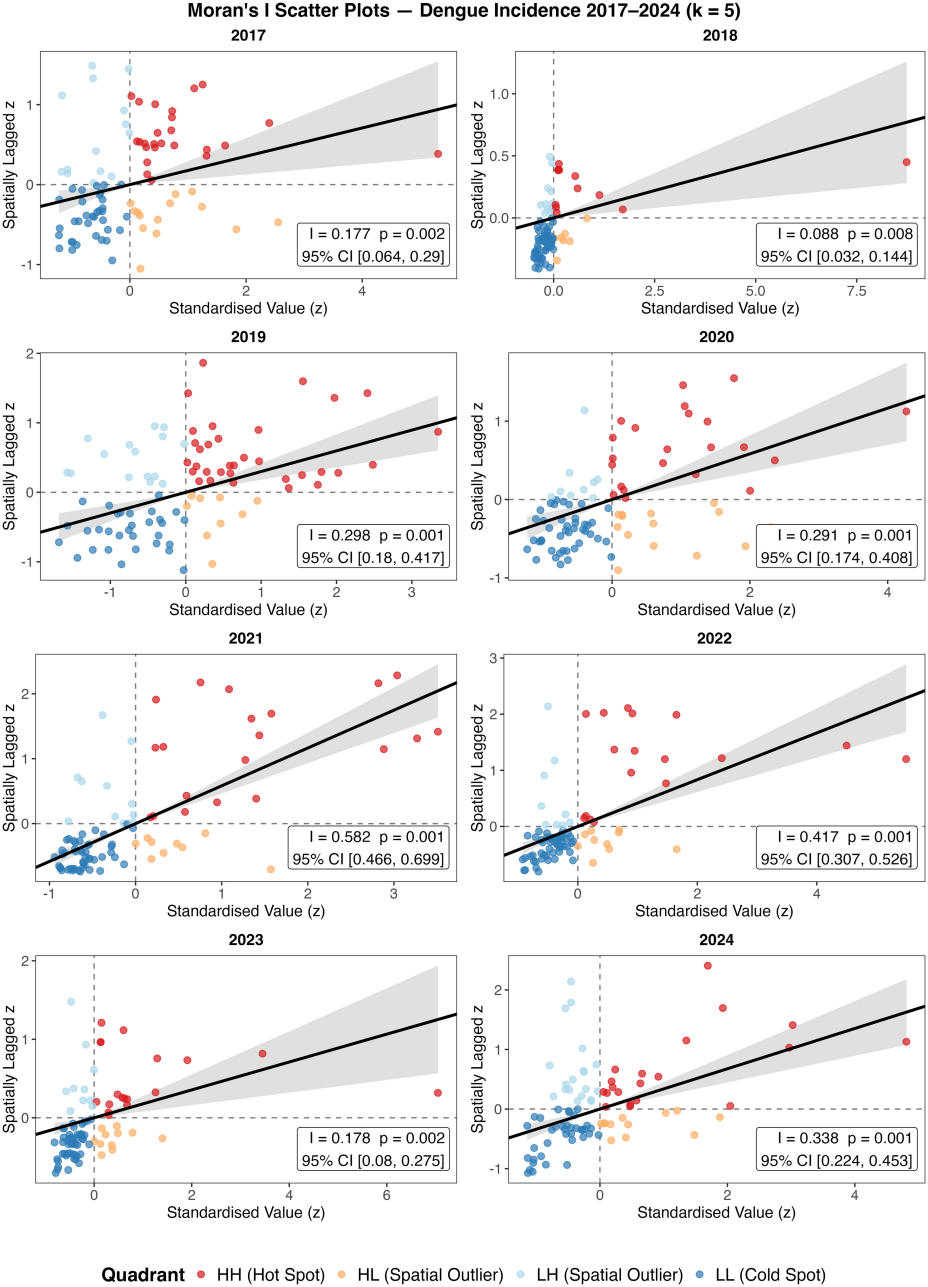
Moran’s I scatter plots from January 2017 to December 2024. A Moran’s I value more than zero indicates positive spatial autocorrelation, I equals zero indicate complete spatial randomness, and negative value indicates dispersion. CI represents confidence interval.

**Figure 3:**
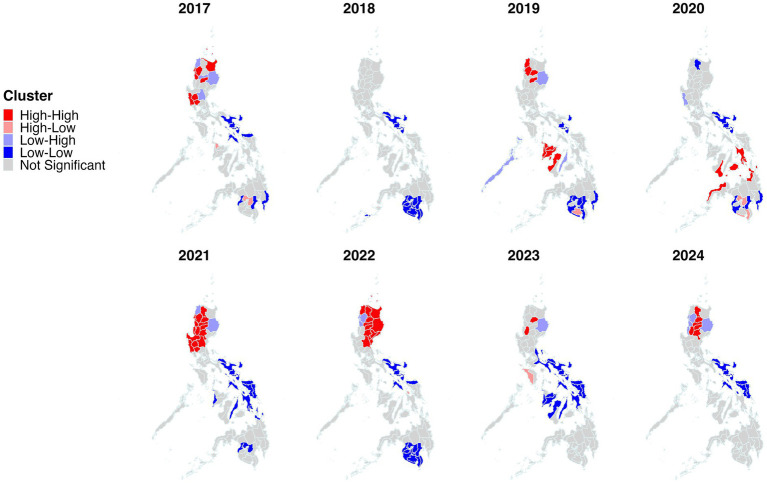
Spatial autocorrelation analysis using local Moran’s I (LISA) for the period January 2017 to December 2024. For cluster analysis, the incidence of each province was used for each respective year. The subnational administrative boundaries of the Philippines were obtained from the United Nations Office for the Coordination of Humanitarian Affairs [available at: https://data.humdata.org/dataset/cod-ab-phl?].

**Figure 4:**
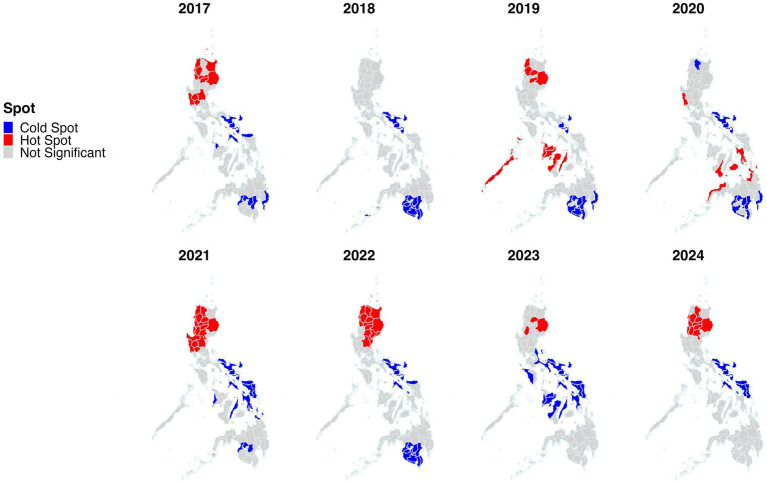
Hotspot analysis of dengue incidence in the Philippines from January 2017 to December 2024 using the Getis-Ord Gi* statistic. The subnational administrative boundaries of the Philippines were obtained from the United Nations Office for the Coordination of Humanitarian Affairs [available at: https://data.humdata.org/dataset/cod-ab-phl?].

The geographical clustering of incidence was investigated by performing spatial autocorrelation and hotspot analyses. Significant clustering (Moran’s *I p* value ≤ 0.05) was identified throughout the study period (Figure 2). Across 2017–2024, Moran’s I was consistently positive (0.088–0.582), indicating persistent spatial clustering of dengue across provinces, but with marked year-to-year variation in strength. Clustering was weakest in 2018 (*I* = 0.088) and 2017/2023 (*I* = 0.177–0.178), increased sharply in 2019–2020 (*I* = 0.298–0.291), peaked in 2021 (*I* = 0.582), then eased in 2022–2023 (*I* = 0.417 to 0.178) before strengthening again in 2024 (*I* = 0.338). Provinces classified as high-high clusters and identified as hotspots by Getis-ord Gi* are listed in Supplementary Table 2. Clusters (high-high) and hotspots were present and varied across the years, with Northern Luzon provinces including Ifugao, Kalinga, Abra, Isabela and Mountain Province being the most frequently identified (Figures 3, 4). Interestingly, many hotspots were observed in 2019. During the 2019 epidemic (51), the disease hotspots were distributed in Northern Luzon, Western and Central Visayas Regions. In Luzon Island, Isabela, Abra, Kalinga, Ilocos Norte and Ifugao provinces were identified. While in the Visayas, provinces of Antique, Negros Occidental, Aklan, Capiz, Iloilo, Guimaras, Palawan, and Cebu were identified as hotspots.

### 3.3 Spatiotemporal clusters

Comparative modeling between discrete Poisson and space–time permutation (STP) models was conducted with varying maximum reported cluster size settings. Table 1 compares the results from the models. Compared with the Poisson model, the STP model detected more and smaller clusters. Interestingly, all the primary clusters identified by the Poisson and STP models are contained within the 2019 period (Table 2). In terms of primary clusters, the STP at the 5% MRCS identified the smallest space–time primary cluster with a 49.66 km spatial radius.

**Table 1:** Number of spatiotemporal clusters identified by the Poisson and space–time permutation (STP) models.

Model	MRCS (%)	No. of clusters	Mean cluster size (km)
Poisson	5	18	68.61
	10	8	98.53
	15	5	222.47
	30	3	318.33
	50	1	397.43
STP	5	23	50.79
	10	10	108.40
	15	8	122.50
	30	3	274.88
	50	3	274.88

**Table 2:** Characteristics of primary clusters of dengue outbreaks based on Poisson and space–time permutation models with various maximum reported cluster size settings.

Model (MRCS %)	Coordinates/Radius (km)	Timeframe	No. of cases	Expected cases	Relative risk	Log likelihood ratio	*p* value
Poisson (5%)	11.369901 N, 122.632934 E/89.41 km	Jun 2019 to Sep 2019	36,894	3,875.17	9.69	50,408.98	<0.001
Poisson (10%)	10.568795 N, 122.614091 E/122.35 km	Jun 2019 to Sep 2019	47,754	6,887.18	7.09	52,047.56	<0.001
Poisson (15%)	9.914284 N, 118.780386 E/505.79 km	Jun 2019 to Sep 2019	65,379	10,951.06	6.15	63,179.245	<0.001
Poisson (30%)	13.209731 N, 123.615752 E/314.28 km	Jul 2019 to Sep 2019	96,507	17,804.63	5.66	86,075.42	<0.001
Poisson (50%)	11.369901 N, 122.632934 E/397.43 km	Jul 2019 to Oct 2019	166,414	39,970.02	4.47	115,310.84	<0.001
STP (5%)	11.369901 N, 122.632934 E/49.66 km	May 2019 to Jul 2019	20,288	4,826.43	–	13,733.55	<0.001
STP (10%)	12.854327 N, 123.928562 E/250.55 km	Mar 2019 to Aug 2019	52,945	21,118.49	–	17,106.07	<0.001
STP (15%)	13.209731 N, 123.615752 E/277.55 km	May 2019 to Sep 2019	81,793	40,001.42	–	17,185.23	<0.001
STP (30%)	13.209731 N, 123.615752 E/289.66 km	May 2019 to Oct 2019	102,597	53,697.03	–	18,178.70	<0.001
STP (50%)	13.209731 N, 123.615752 E/289.66 km	May 2019 to Oct 2019	102,597	53,697.03	–	18,178.70	<0.001

**Figure 5:**
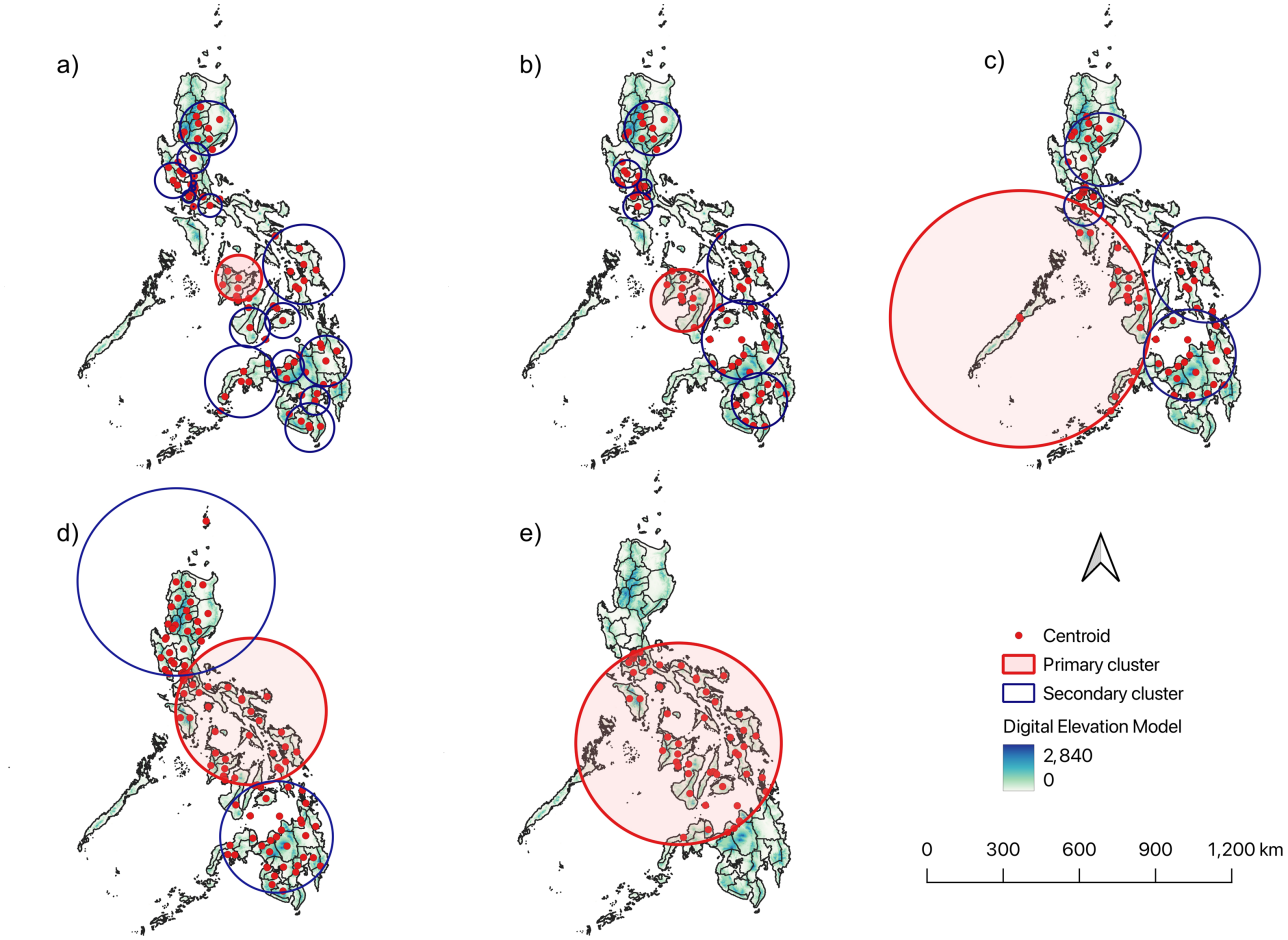
Spatiotemporal cluster detection of dengue cases in the Philippines from January 2017 to December 2024 using SaTScan Poisson model with varying maximum reported cluster size (MRCS) including: **(a)** 5%; **(b)** 10%; **(c)** 15%; **(d)** 30%; and **(e)** 50%. The red circle represents the primary cluster, while the blue circle represents the secondary cluster. The subnational administrative boundaries of the Philippines were obtained from the United Nations Office for the Coordination of Humanitarian Affairs [available at: https://data.humdata.org/dataset/cod-ab-phl?].

**Figure 6:**
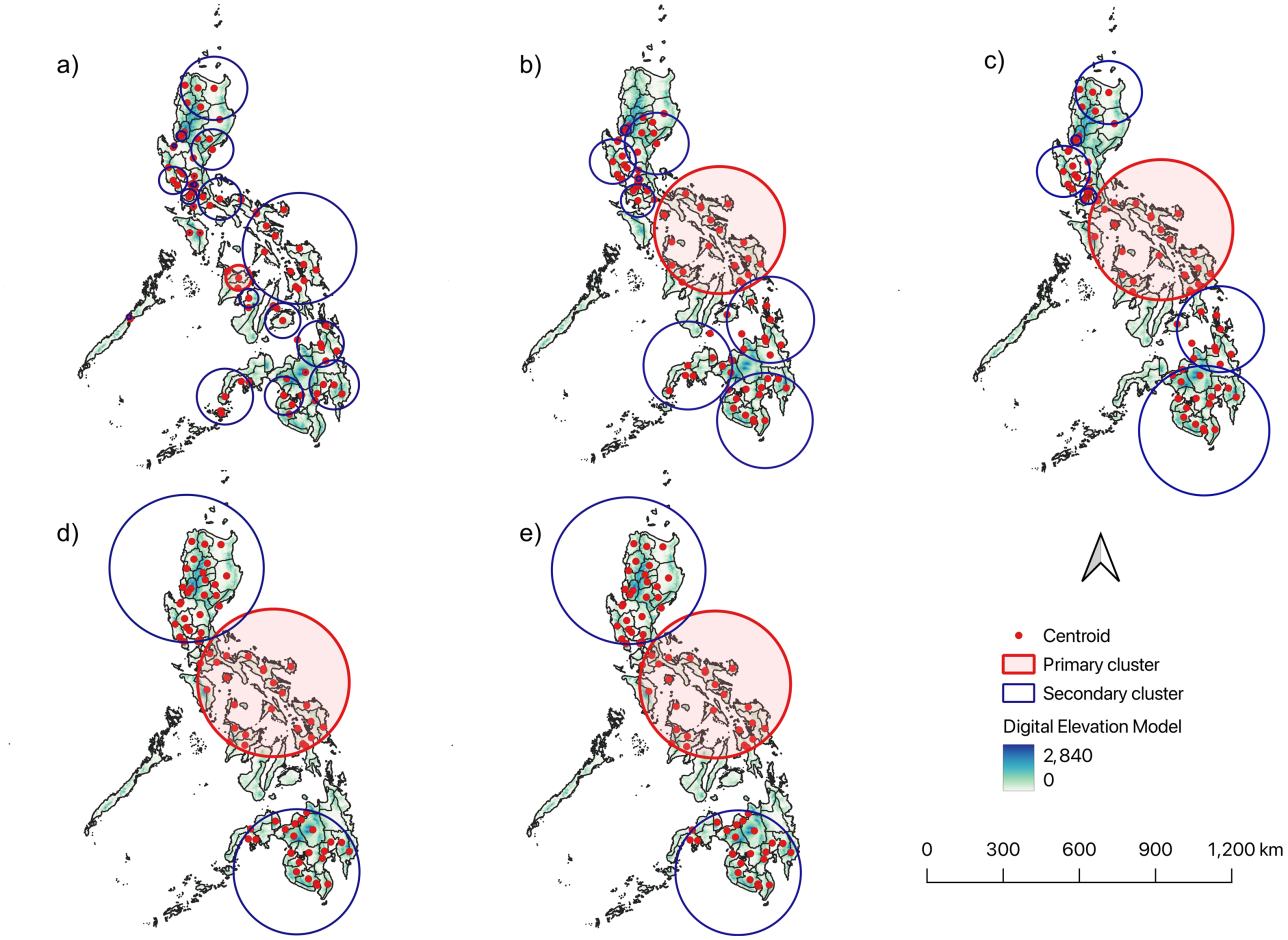
Spatiotemporal cluster detection of dengue cases in the Philippines from January 2017 to December 2024 using SaTScan space–time permutation model (STP) with varying maximum reported cluster size (MRCS) including **(a)** 5%, **(b)** 10%, **(c)** 15%, **(d)** 30%, and **(e)** 50%. The red circle represents the primary cluster, while the blue circle represents the secondary cluster. The subnational administrative boundaries of the Philippines were obtained from the United Nations Office for the Coordination of Humanitarian Affairs [available at: https://data.humdata.org/dataset/cod-ab-phl?].

The primary cluster was identified by both the Poisson and the STP spatiotemporal models. The Poisson model identified 17 secondary clusters at 5% MRCS, seven secondary clusters at 10% MRCS, four at 15% MRCS, and two secondary cluster at 30% MRCS, with no secondary clusters detected at the 50% MRCS setting (Figure 5). The STP models, on the other hand, identified 22 secondary clusters at 5% MRCS, nine secondary clusters at 10% MRCS, seven secondary clusters at 15% MRCS, and two secondary clusters at both 30 and 50% MRCS (Figure 6). The 30 and 50% MRCS STP models have the same results.

## 4 Discussion

The incidence of dengue continues to increase in the Philippines, a dengue-endemic country. At present, understanding the spatiotemporal epidemiology of dengue at the national scale is crucial. The study aimed to identify spatial patterns and spatiotemporal clustering of dengue incidence in the Philippines. Results of the study show high concentration of cases was consistently observed in the National Capital Region. Significant clustering is demonstrable throughout the study period with clusters and hotspots’ locations shifting from year to year. Persistent clusters were identified in Northern Luzon, specifically the provinces of Ifugao, Kalinga, Abra, Isabela and Mountain Province. Spatiotemporal models consistently revealed areas within the Western Visayas region as primary clusters. Changing MRCS to lower values led to identification of smaller but more clusters. These findings highlight the importance of refining spatiotemporal models to achieve meaningful results for targeted control implementation.

As an endemic country, the occurrence of dengue in the Philippines is widespread, with all the provinces being highly affected. Notably, a high number of cases were consistently concentrated in NCR during the study period. The region comprises densely populated cities, such as the City of Manila and Quezon City. Factors such as high population density, microclimatic conditions, and rapid mobility predispose major cities to be more affected by dengue, particularly in endemic countries (1). Considering the population of the region, the incidence of dengue per 100,000 people decreased in 2020 and 2021, during the COVID-19 pandemic. It was hypothesized that, during this period, restrictions on internal movement, public transport closures, fear of contracting COVID-19, and increased hesitancy to seek care or report illness at health facilities were associated with reduced dengue incidence rates (25, 52). Furthermore, the redirection of healthcare resources toward COVID-19 response contributed to gaps in the reporting and surveillance of other communicable diseases. This situation was compounded by changes in mobility patterns and shifts in health priorities, which affected timely interventions and data completeness for diseases like dengue. Studies have documented significant underreporting of dengue and other vector-borne diseases during 2020 and 2021, highlighting the impact of the pandemic on routine disease surveillance and reporting systems in the Philippines (53). Additionally, lockdowns and social distancing policies altered transmission dynamics and delayed dengue control measures, further complicating surveillance accuracy (54). Nonetheless, the NCR still contributes greatly (~11%) to the frequency of cases in the country, which requires strengthening vector control enforcement, particularly in major cities.

The incidence of dengue in the Philippines varies with time and geographic area. Spatial autocorrelation and hotspot analysis reveal distinct incidence clusters every year. The observed variability in high-risk areas underscores the necessity for prompt annual surveillance data collection and analysis to effectively direct focused intervention strategies (9). Medina et al. (9) added that collection and analysis in real-time should be incorporated in the early warning system to detect emerging hotspots in a given year, aiding public health authorities’ timely response. This further suggests the importance of incorporating prospective spatiotemporal cluster detection in the surveillance system, which can be performed via spatial scan statistics or SaTScan (46, 55). Notably, the provinces of Ifugao, Kalinga, Abra, Isabela and Mountain Province have been identified as hotspots more frequently than other provinces. The persistence of significant spatial autocorrelation suggests that dengue risk in the Philippines is not randomly distributed but repeatedly concentrates in provinces with shared underlying determinants. Recurrent clustering is plausibly driven by spatially structured factors such as high population density and rapid urbanization, which increase human–vector contact and breeding sites (56, 57); rainfall and temperature patterns that influence *Aedes* mosquito abundance and seasonality (58, 59); and interprovincial and interisland travel, which facilitates virus movement between connected population centers (60). These mechanisms imply that the identified hotspots likely reflect enduring transmission ecology and connectivity, rather than isolated outbreaks. Furthermore, these Northern Luzon provinces must be considered by authorities in prioritizing fund allocation for vector-borne disease control and prevention.

Poisson and STP models were utilized to determine spatiotemporal clusters with different MRCS settings. Such approach is with the presumption that deciding the most appropriate pattern could be difficult if only based on a single spatiotemporal model’s results (49). Therefore, performing multiple spatiotemporal analytical approaches with varying parameters, including adjustments to configuration, scale, and concentration of case distributions is crucial, rather than relying on a single fixed parameter set when identifying disease clustering patterns (61, 62). The models revealed that the primary cluster of dengue incidence included areas within the Western Visayas region, whereas most of the secondary clusters were detected in other areas of the country. In 2019, most of dengue hotspots were detected in this region (Figures 3, 4) with the Iloilo province recording the highest number of cases nationwide. The most likely cluster has an estimated relative risk of 9.69, indicating that dengue incidence inside the cluster was approximately 9.7-fold higher than in the rest of the study area during the cluster period. For local health offices, this supports prioritizing hotspot provinces for earlier and more intensive surveillance, targeted vector control, and pre-positioning of clinical and response resources especially in the high-risk months. Furthermore, the region should bolster long-term vector control at a large scale and strengthen health systems to manage excessive dengue cases, especially when epidemics or adverse climatic conditions are forecasted to occur in the following year. In addition, future vaccination may be considered a sustainable option for disease control in the region (63); however, further studies must be performed to understand the current acceptability of dengue vaccines in the region and how to address vaccine hesitancy (64).

Spatiotemporal analyses reveal primary clusters with a wide range of spatial radii (49.66–505.79 km), which are likely provincial to regional in scope. Regional dengue hotspots are associated with improper water systems and inefficient or lack waste management systems in highly urbanized areas (65, 66). Iloilo City is the fourth most highly urbanized city in the Philippines, following the City of Manila (32), which is among the localities with the highest incidence of dengue (mean ± SD = 21.65 ± 58.24 per 100,000 people) in the Western Visayas region. Urbanization in highly urbanized Philippine cities is associated with deteriorating water bodies from inadequate treatment despite high water use, aging infrastructure, ineffective waste management, and poor waste segregation (e.g., plastic containers mixed with liquid waste) (67–69). These conditions increase stagnant water and enhance *Aedes* breeding conditions (70). Another reason for the increased incidence in the region is the shortage of water supply. In Western Visayas, water shortages also drive household water storage especially during recent El Niño events unintentionally creating additional mosquito breeding sites (71). This is also documented in Venezuela, where households store extra water during summer, especially in rural areas, contributing to dengue incidence by providing more mosquito breeding habitats (72). The wide spatial extent of the primary clusters indicates that dengue control should be prioritized at provincial-to-regional scales through coordinated surveillance and response. Because hotspots are linked to inadequate water and waste management in highly urbanized areas, resources should be focused on urban centers in Western Visayas, particularly Iloilo City, which shows high dengue incidence.

The Philippine intervention toward mosquito-borne diseases mainly involves integrated vector control, a combination of health education, environmental sanitation, and community mobilization (68). DOH emphasizes the 4S strategy, which includes searching for and destroying mosquito breeding grounds by eliminating stagnant water and its containers; self-protection measures such as using insect repellents and wearing long-sleeved clothing; seeking early consultation when experiencing symptoms such as fever, headache, body pain, or skin rashes; and supporting fogging or spraying in local hotspot areas (67). However, these control efforts are hampered by stakeholders’ lack of responsibility for dengue prevention (69). The above findings imply that authorities should focus not only on vector control but also on the improvement of community water systems and waste management systems. The reinforcement of policies on waste management is highly needed.

The effects of different spatiotemporal parameter settings on the results were examined in this study. Setting the MRCS to lower values resulted in smaller but more clusters. This finding indicates that adjusting the MRCS to lower values could further define the location of clusters and are more meaningful than clusters identified via the default settings in the study. Notably, both the Poisson and the STP models were concordant on the identified primary cluster and all the clusters’ occurrence periods, signifying the peaks of the outbreak in 2019. Varying MRCS setting values were applied in this study over the maximum scanning window size (MSWS) because altering the MSWS leads to statistical issues related to multiple comparisons. The maximum scanning window size parameter varies in some studies (73). However, conducting the analysis multiple times could potentially introduce multiple comparison and statistical issues (47, 49, 73). Hence, it is suggested to use the default MSWS while the MRCS may be varied (47, 49). The approach of varying MRCS has also been applied in other fields of research (74, 75). The results from different MRCS settings provide varying scenarios that can be further explored to determine their relevance to specific objectives. Some objectives may align better with larger cluster results, whereas others may prioritize smaller clusters (55). For example, if authorities aim to capture broader spatiotemporal clustering, a larger maximum reported cluster size (MRCS) may be used (e.g., 50%). Conversely, if the goal is to detect more localized clusters, such as when the DOH had to implement control measures at the village level, smaller MRCS values are preferable. When selecting for the appropriate MRCS value, the research objective and the availability of resources should be considered.

The findings of the study support shifting from broadly applied interventions to risk-based operations guided by the hotspot and SaTScan cluster maps. These maps can be integrated into an early warning system by designating repeatedly affected provinces as priority sentinel areas and applying monthly alert triggers (e.g., cases exceeding the province’s seasonal baseline for ≥2 consecutive months, or detection of a significant space–time cluster) that activate pre-defined response actions. In practice, triggers could prompt intensified community source reduction and risk communication, targeted entomological checks in high-incidence municipalities, and rapid coordination with local facilities. Cluster outputs can also guide preparedness logistics by identifying where to pre-position clinical supplies and response capacity ahead of peak months, including diagnostics and treatment consumables, and by positioning provincial/regional surge teams for faster deployment to flagged areas. Finally, “area-based surveillance” in a monthly system can be implemented by routinely reviewing municipal (or barangay, if available) contributions within hotspot provinces, prioritizing investigation of localities driving monthly increases, and using these sub-provincial signals to target control activities rather than distributing efforts uniformly.

To the best of our knowledge, this is the first study in the Philippines to apply spatiotemporal cluster analysis to dengue incidence via spatial scan statistics at the national scale. This study addresses the current gap in knowledge concerning the spatiotemporal clustering of dengue at the national level. The study differs from previous studies in that it utilized more recent data, covered a longer period and had a wider geographic scope. The spatiotemporal models applied in this study offer complementary insights that are relevant across different local contexts. The discrete Poisson model is most appropriate when reliable population denominators are available, as it identifies clusters of elevated risk relative to the population at risk and can inform resource allocation based on estimated excess incidence. In contrast, the space–time permutation model is particularly valuable in data-limited settings, since it relies only on case locations and times and can still detect unusual space–time aggregations when population estimates are unavailable, uncertain, or rapidly changing. In addition, the method employed herein did not rely solely on the default settings since it may result in clusters that are too large, potentially diminishing significance. The analysis was also based on advanced settings rather than basic settings. This approach presents different scenarios to better accommodate the varying needs of health authorities and stakeholders. To achieve meaningful results, public health practitioners may apply smaller MRCSs when investigating local outbreaks and larger MRCSs when aiming for a larger geographic scope.

However, the study has certain limitations. Point pattern analysis was not possible because the exact location of cases was untraceable. Therefore, centroids were used for spatiotemporal modeling, as applied in other studies (20, 76). This centroid approximation may not reflect the true residential locations of cases and can mask within-area heterogeneity (modifiable areal unit problem). Consequently, some detected clusters may have relatively large spatial radii. These radii should be interpreted as the size of the SaTScan scanning window that best fits the aggregated data, rather than the actual geographic footprint of transmission. The analysis was performed with provinces and major cities as units because of data limitations. Interpretation of the study findings should be made with caution because dengue case counts derived from passive surveillance systems are susceptible to under-reporting. In addition, the spatiotemporal models used in the study based on a spatial scan-statistics framework did not explicitly adjust for several potentially important confounding factors that may influence dengue transmission and the observed spatiotemporal patterns. Future studies should incorporate these covariates and evaluate the robustness of the observed patterns using alternative modeling approaches, such as maximum entropy or Bayesian hierarchical models. Furthermore, owing to the scale of the study and data restrictions, the retrieval of demographic or socioeconomic data from each case was not possible. This has limited our ability to identify the factors that drive the spatiotemporal patterns and trends of dengue found in this study, which warrants further investigation. Future work may consider using geocoded addresses that would allow point pattern analyses which may reveal micro-scale hotspot structures or finer units (municipal or village) with longer time periods for deeper spatial and temporal analysis. Despite these limitations, varying the MRCS represents a sensitivity analysis that evaluate the stability of the reported significant clusters under different reporting thresholds. The primary clusters remained stable across MRCS settings which supports the robustness of the main findings. The findings of the study still further the understanding of the spatiotemporal epidemiology of dengue in the Philippines.

## 5 Conclusion

This study aims to understand the spatiotemporal epidemiology of dengue in the Philippines. To our knowledge, this is the first study in the Philippines to apply spatiotemporal cluster analysis via spatial scan statistics at the national scale. The spatial clusters varied by year, with the provinces of Ifugao, Kalinga, Abra, Isabela and Mountain Province being the most frequently identified hotspots. Comparative spatiotemporal models revealed areas within the Western Visayas region as under the primary cluster. The application of varying MRCS has been demonstrated to effectively detect meaningful clusters. Thus, health agencies and authorities in the Philippines could adopt these approaches to further characterize disease epidemiology, particularly in terms of spatial and spatiotemporal clustering. These methods can also be integrated into regional or provincial surveillance and early warning systems to guide timely implementation of targeted interventions.

## Data Availability

The original contributions presented in the study are included in the article/Supplementary material, further inquiries can be directed to the corresponding author.
