# Supplementary material for: Spatiotemporal clusters and dengue hotspots in the Philippines: a nationwide analysis spanning 2017–2024
Source: Front Public Health. 2026 Apr 22;14:1781800. doi: 10.3389/fpubh.2026.1781800 (PMC13143918; doi:10.3389/fpubh.2026.1781800)
Supplement: Supplementary file 1 [file Supplementary_file_1.docx]

Supplementary Material

# Supplementary Tables and Figures

## Supplementary Tables

**Supplementary Table 1**. Significant clusters and hotspots.

| **Year** | **Moran’s I** | **Getis-Ord Gi*** | |
| --- | --- | --- | --- |
| 2017 | Pampanga 0.012  Ifugao 0.017  Cagayan 0.023  Ilocos Sur 0.030  Abra 0.030  Tarlac 0.047  Zambales 0.047 | Isabela  Mountain Province  Pampanga  Ilocos Norte  Ifugao  Cagayan  Nueva Ecija  Batanes  Ilocos Sur  Abra  Tarlac  Zambales | 0.006  0.006  0.012  0.014  0.017  0.023  0.027  0.029  0.03  0.03  0.047  0.047 |
| 2018 | Batanes 0.009 | Batanes | 0.009 |
| 2019 | Antique 0.001  Negros Occidental 0.001  Aklan 0.002  Capiz 0.002  Iloilo 0.003  Guimaras 0.019  Abra 0.021  Kalinga 0.028  Ilocos Norte 0.029  Ifugao 0.046 | Antique  Negros Occidental  Aklan  Capiz  Iloilo  Guimaras  Isabela  Abra  Palawan  Kalinga  Ilocos Norte  Ifugao  Cebu | 0.001  0.001  0.002  0.002  0.003  0.019  0.021  0.021  0.025  0.028  0.029  0.046  0.049 |
| 2020 | Bohol 0.002  Camiguin 0.002  Siquijor 0.003  Southern Leyte 0.013  Surigao del Norte 0.019  Negros Oriental 0.022  Leyte 0.033  Zamboanga del Norte 0.039  Agusan del Norte 0.047 | Bohol  Camiguin  Siquijor  Southern Leyte  Zambales  Surigao del Norte  Negros Oriental  Leyte  Zamboanga del Norte  Agusan del Norte | 0.002  0.002  0.003  0.013  0.014  0.019  0.022  0.033  0.039  0.047 |
| 2021 | Ilocos Sur 0.001  Pangasinan 0.001  Abra 0.001  Ifugao 0.001  Mountain Province 0.001  La Union 0.002  Zambales 0.002  Pampanga 0.003  Kalinga 0.003  Benguet 0.005  Tarlac 0.012  Nueva Vizcaya 0.017  Apayao 0.018  Nueva Ecija 0.025 | Ilocos Sur  Pangasinan  Abra  Ifugao  Mountain Province  La Union  Zambales  Isabela  Pampanga  Kalinga  Benguet  Tarlac  Ilocos Norte  Nueva Vizcaya  Apayao  Nueva Ecija | 0.001  0.001  0.001  0.001  0.001  0.002  0.002  0.003  0.003  0.003  0.005  0.012  0.013  0.017  0.018  0.025 |
| 2022 | Ilocos Norte 0.001  Isabela 0.001  Cagayan 0.002  Batanes 0.003  Ifugao 0.004  Kalinga 0.004  Quirino 0.011  Apayao 0.012  Aurora 0.015  Mountain Province 0.015  Nueva Vizcaya 0.028  Nueva Ecija 0.043 | Ilocos Norte  Isabela  Cagayan  Abra  Batanes  Ifugao  Kalinga  Quirino  Apayao  Aurora  Mountain Province  Ilocos Sur  Nueva Vizcaya  Nueva Ecija | 0.001  0.001  0.002  0.002  0.003  0.004  0.004  0.011  0.012  0.015  0.015  0.02  0.028  0.043 |
| 2023 | Benguet 0.038  Kalinga 0.047 | Isabela  Benguet  Kalinga | 0.017  0.038  0.047 |
| 2024 | Ifugao 0.001  Kalinga 0.001  Benguet 0.004  Mountain Province 0.004  Nueva Vizcaya 0.014  Apayao 0.021 | Isabela  Ifugao  Kalinga  Ilocos Sur  Abra  Benguet  Mountain Province  Nueva Vizcaya  Apayao  La Union | 0.001  0.001  0.001  0.002  0.003  0.004  0.004  0.014  0.021  0.029 |

## Supplementary Figures


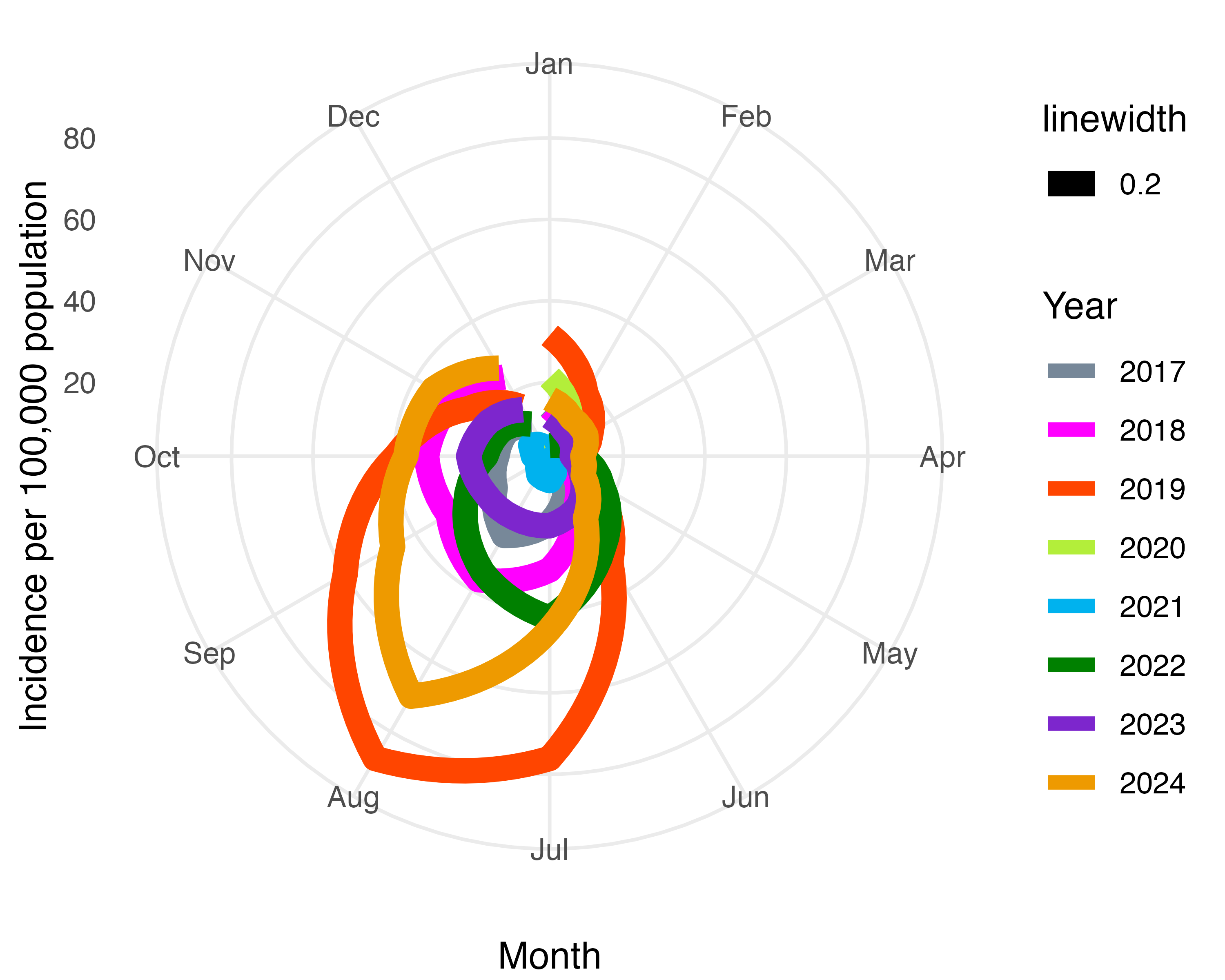


**Supplementary Figure 1.** Seasonal plot of mean dengue incidence in the Philippines from January 2017-December 2024.


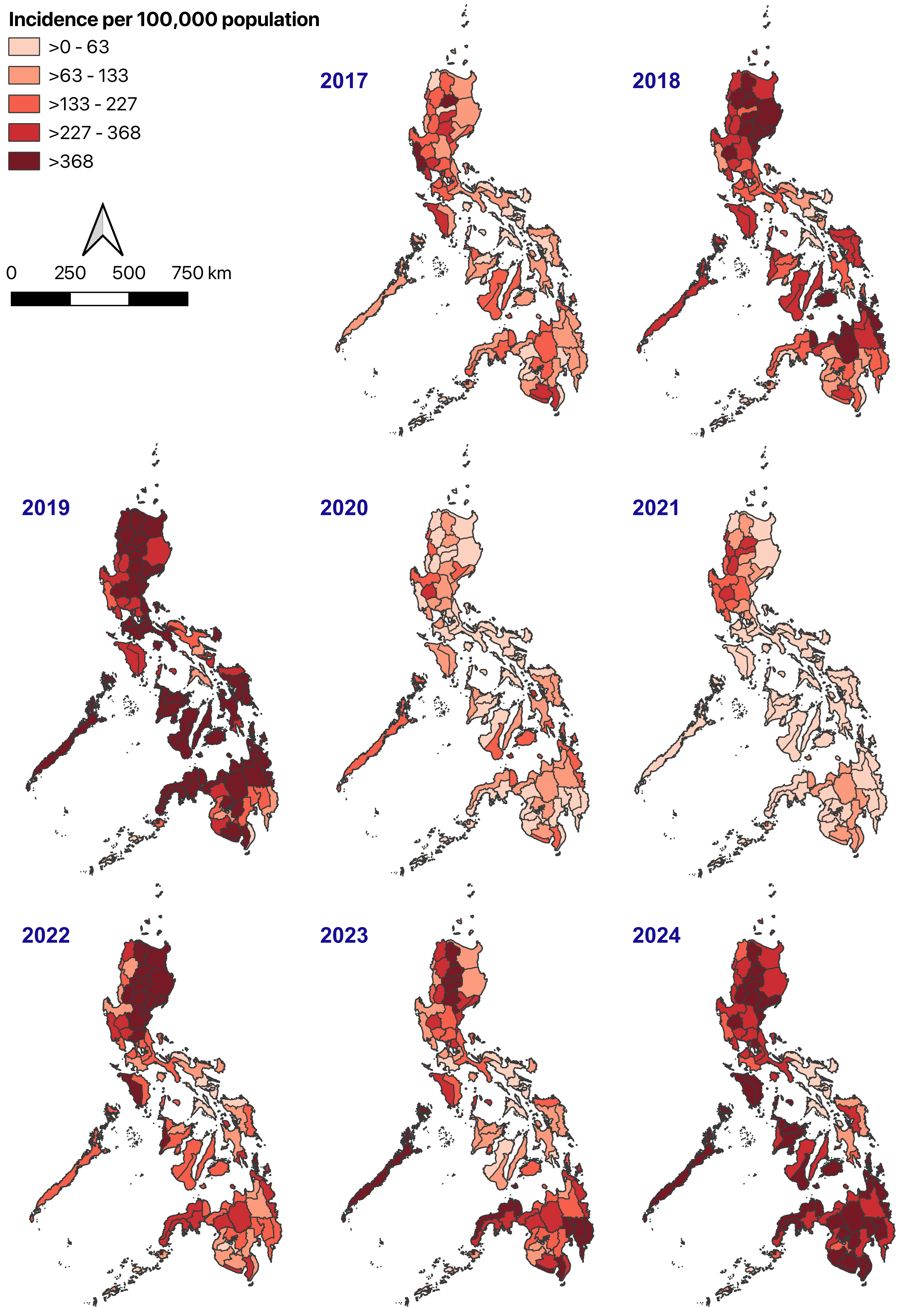


**Supplementary Figure 2.** Choropleth map of dengue incidence in the Philippines from January 2017-December 2024.


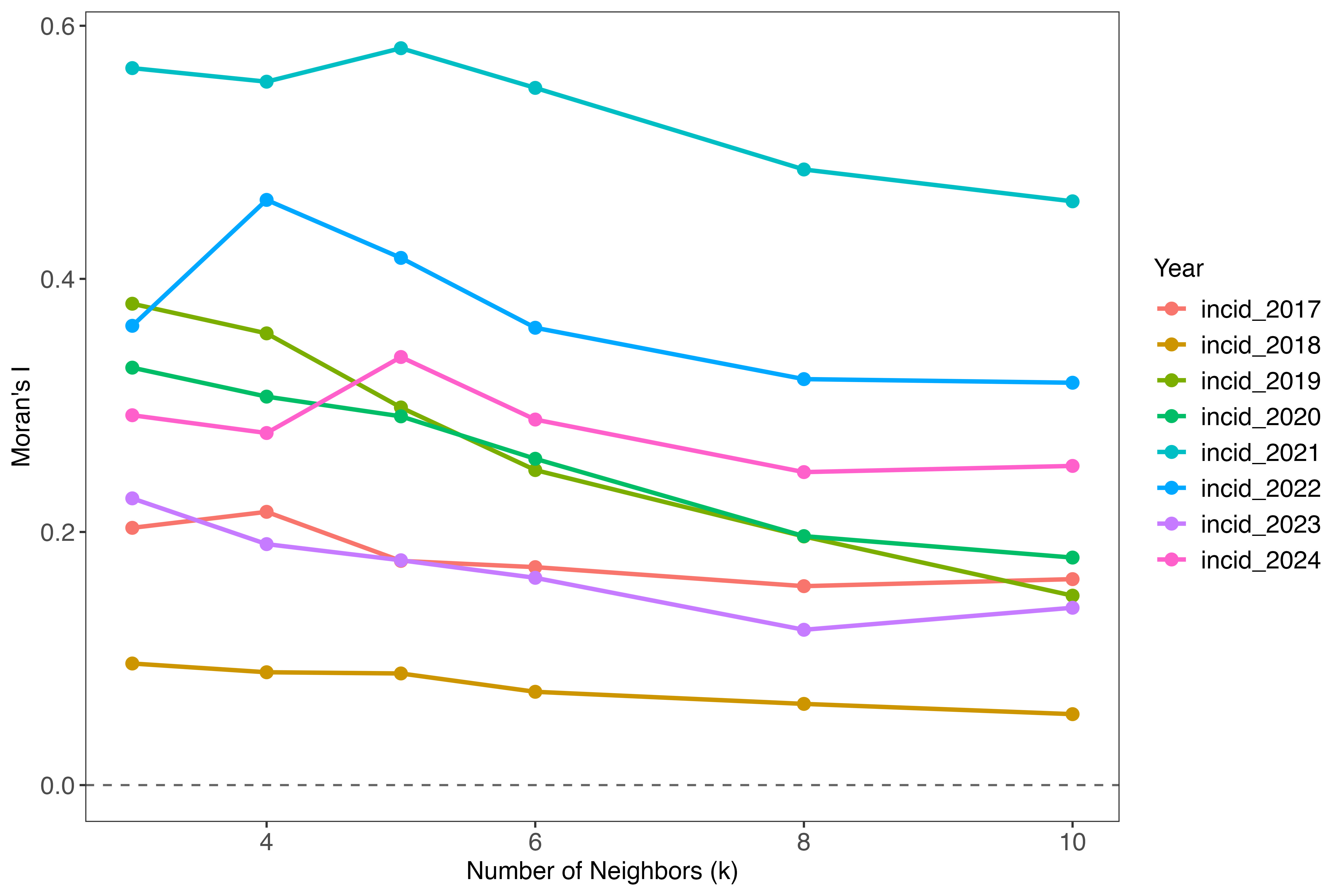


**Supplementary Figure 3.** Global Moran’s I sensitivity analysis. Different k values of kNN was applied in generating spatial weights for dengue incidence from 2017 – 2024.


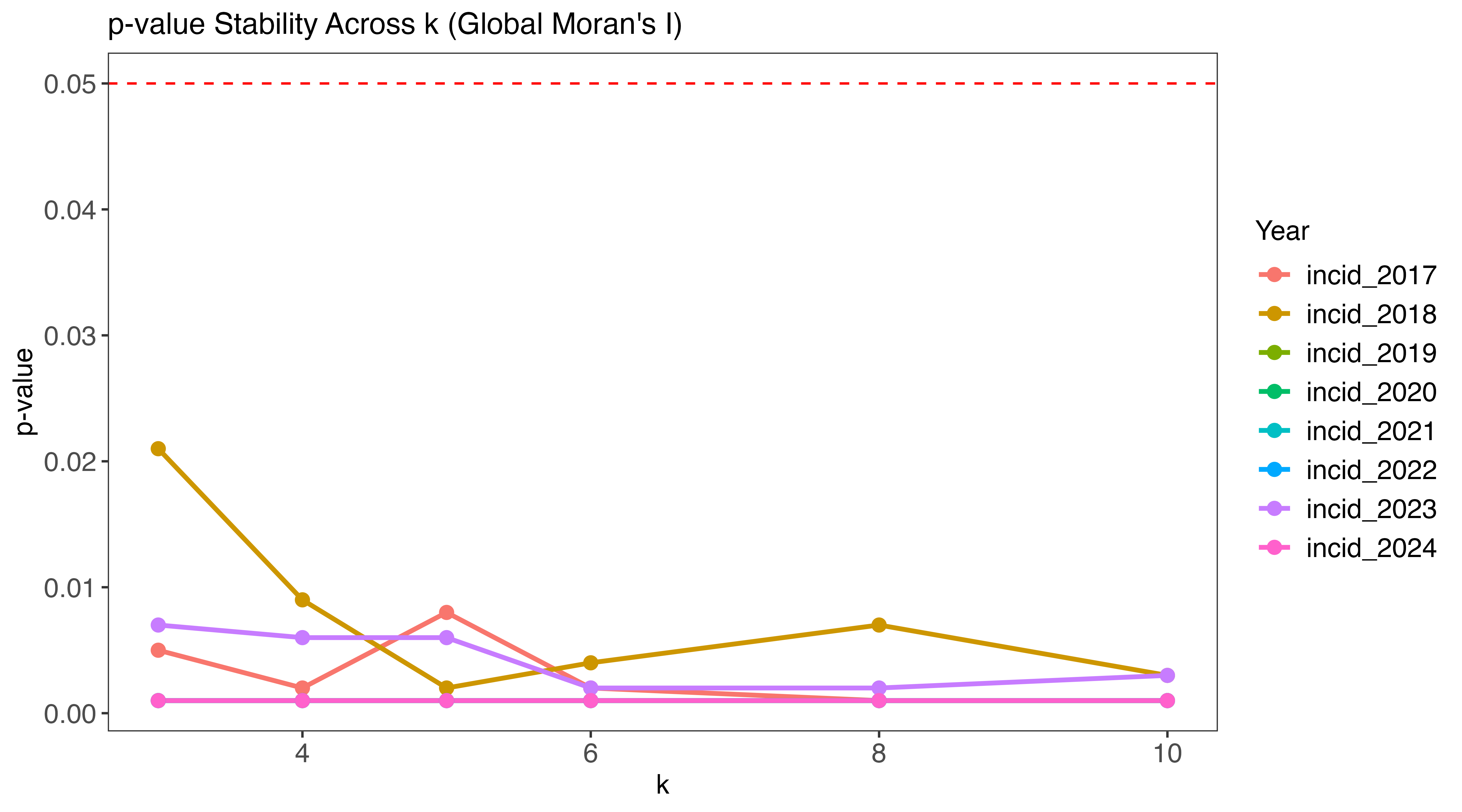


**Supplementary Figure 4.** Global Moran’s I p-value by different k values from 2017 – 2024.


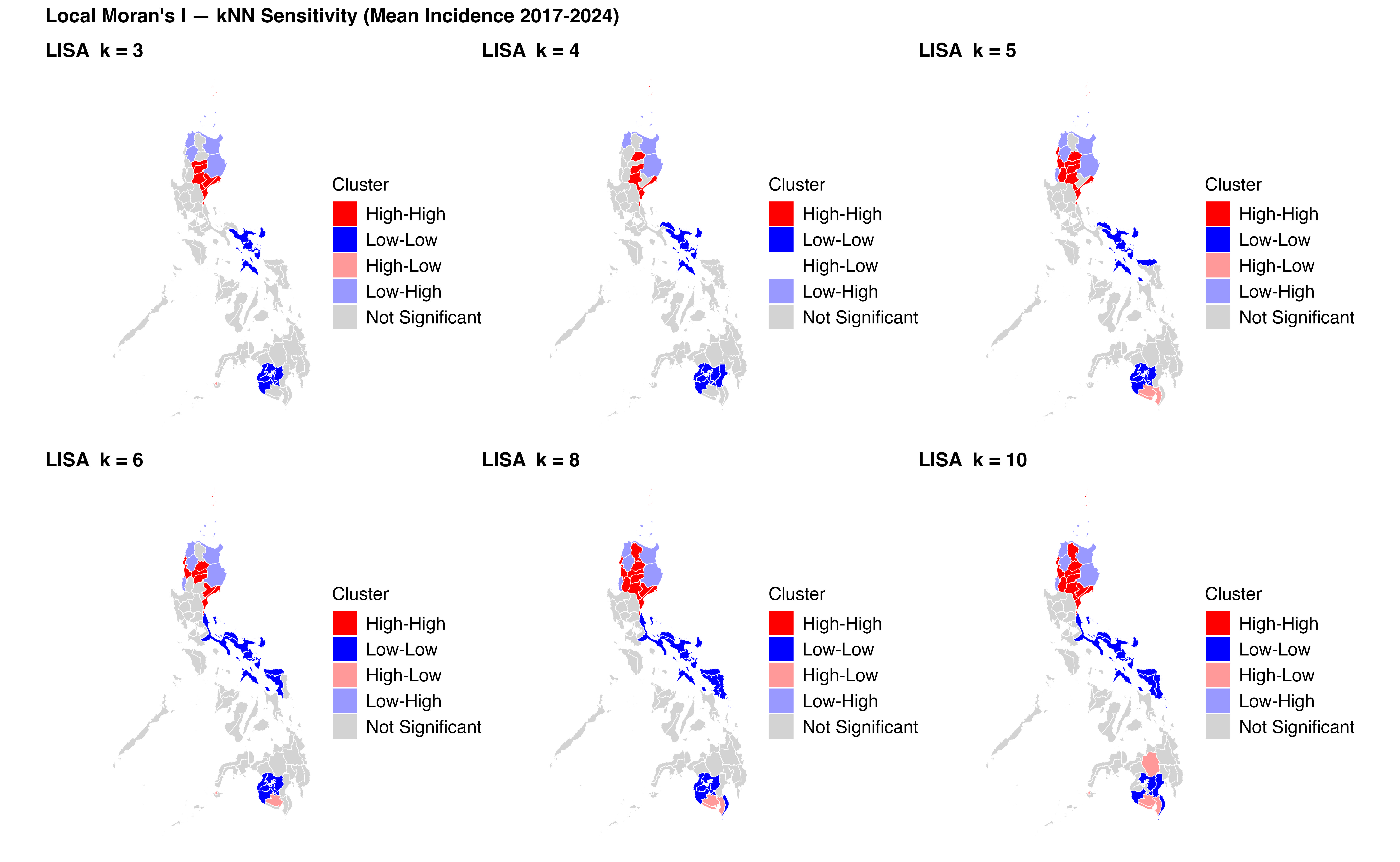


**Supplementary Figure 5.** Local Moran’s sensitivity analysis using varying k values and mean incidence from 2017 – 2024.


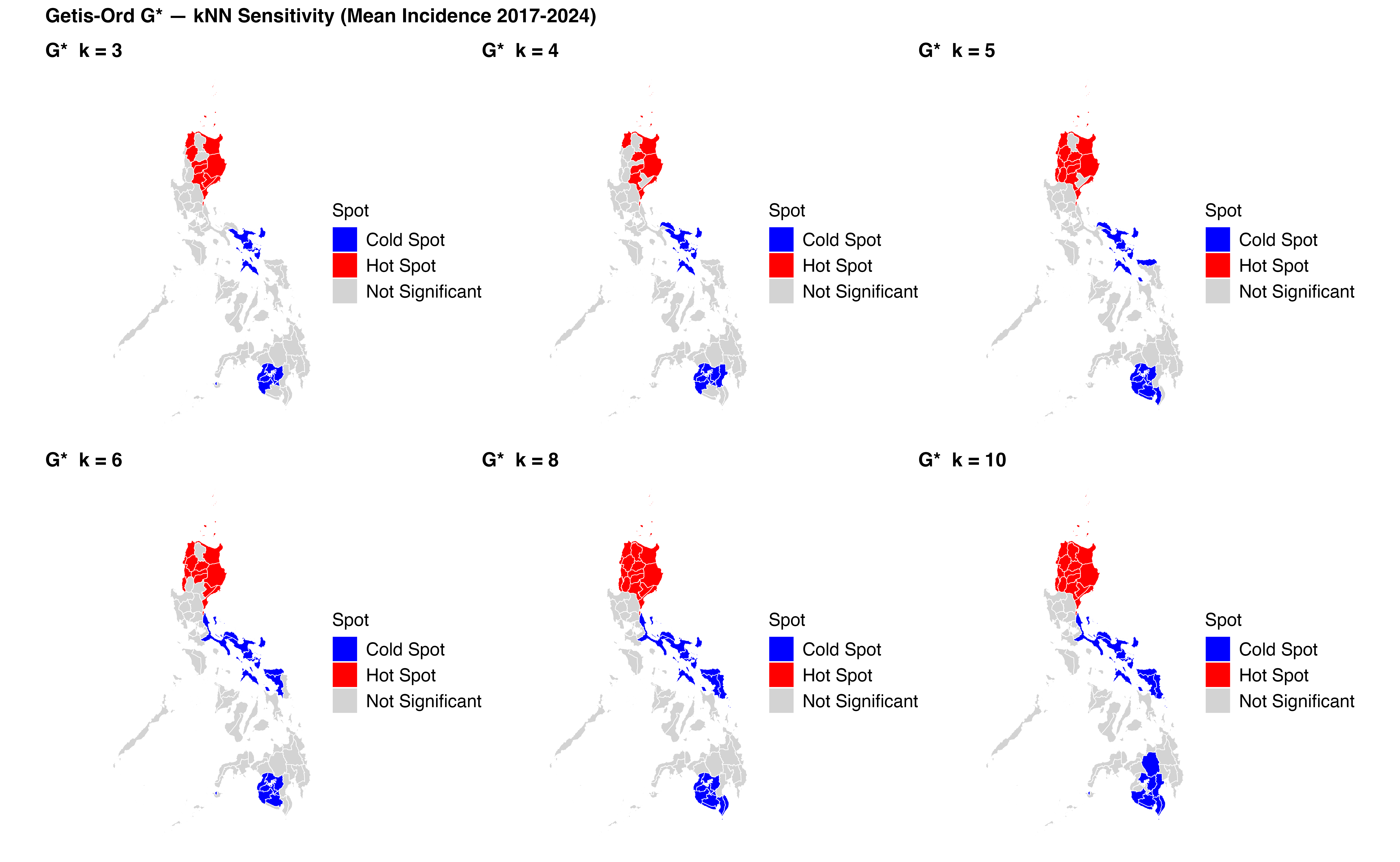


**Supplementary Figure 6.** Getis-Ord-G* sensitivity analysis using varying k values and mean incidence from 2017 – 2024.


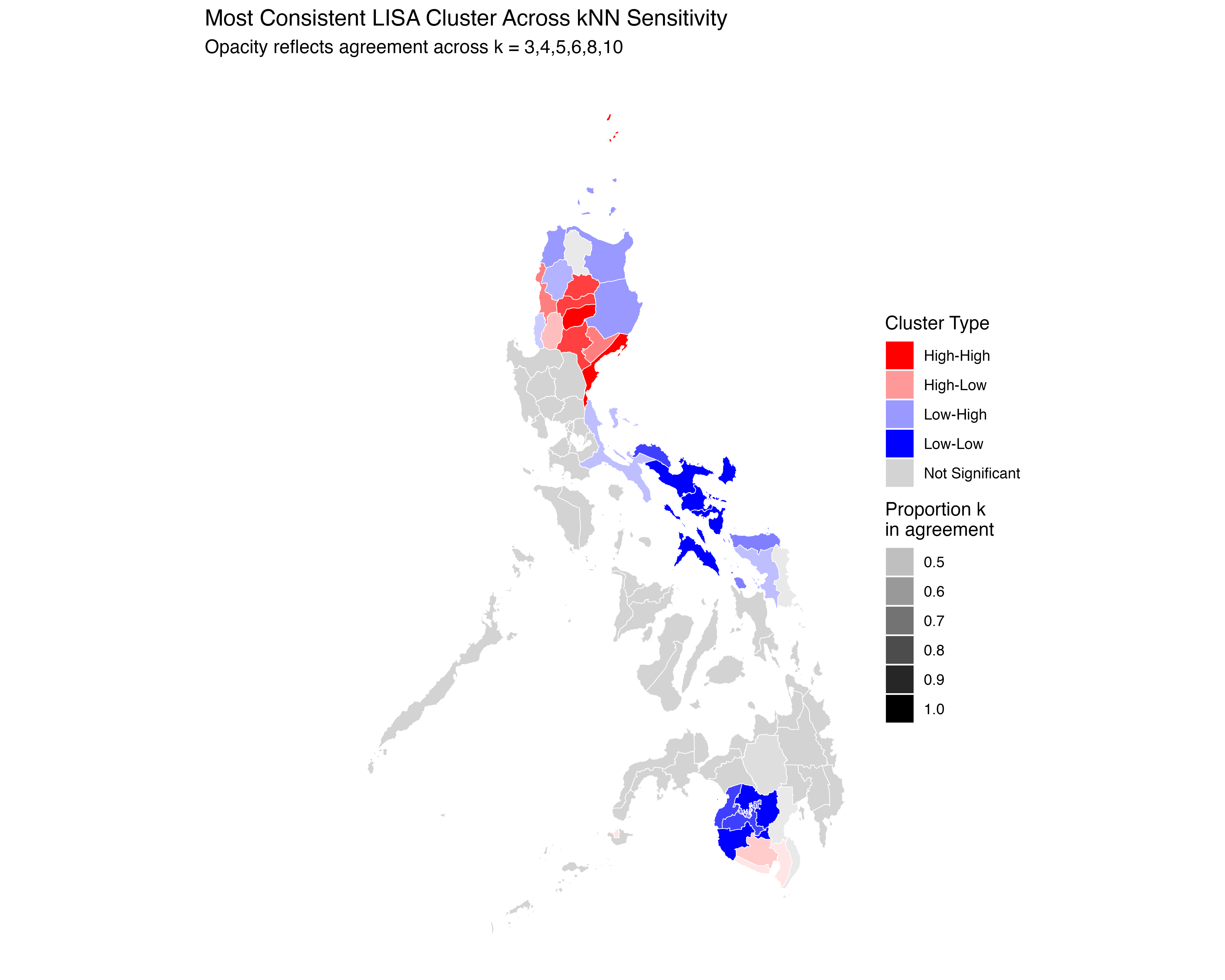


**Supplementary Figure 7.** LISA agreement map of clusters from sensitivity analysis of varying k applied on mean dengue incidence.
