# Supplementary material for: Spatiotemporal clusters and dengue hotspots in the Philippines: a nationwide analysis spanning 2017–2024
Source: Front Public Health. 2026 Apr 22;14:1781800. doi: 10.3389/fpubh.2026.1781800 (PMC13143918; doi:10.3389/fpubh.2026.1781800)
Supplement: Supplementary file 2 [file Supplementary_file_2.docx]

Supplementary Material

# Supplementary Codes

## Spatial Autocorrelation, Hotspot and Sensitivity Analysis

# ============================================================

# INSTALL/LOAD SPDEP FOR GLOBAL MORAN'S I

# rgeoda 0.1.1 has no global_moran() — use spdep instead

# ============================================================

install.packages("spdep") # run once if not installed

library(rgeoda)

library(spdep)

library(sf)

library(ggplot2)

library(dplyr)

library(patchwork)

# ============================================================

# 1. LOAD & PREP DATA

# ============================================================

shp <- st_read("Incidence_geoda.shp")

shp <- st_make_valid(shp)

print(names(shp)) # confirm column names

# Define incidence variables (adjust if needed)

inc_vars <- names(shp)[grepl("2017|2018|2019|2020|2021|2022|2023|2024",

names(shp), ignore.case = TRUE)]

print(inc_vars)

# Convert to numeric

for (v in inc_vars) shp[[v]] <- as.numeric(shp[[v]])

# Mean incidence

shp$Mean_Inc <- rowMeans(st_drop_geometry(shp[, inc_vars]), na.rm = TRUE)

k_values <- c(3, 4, 5, 6, 8, 10)

k_opt <- 5 # chosen after sensitivity analysis

# ============================================================

# 2. WEIGHT CONVERSION HELPER

# rgeoda knn_weights -> spdep listw (needed for global Moran's I)

# ============================================================

rgeoda_knn_to_listw <- function(data_sf, k) {

# Build knn via spdep using centroids (mirrors rgeoda knn logic)

coords <- st_coordinates(st_centroid(st_geometry(data_sf)))

knn <- knearneigh(coords, k = k)

nb <- knn2nb(knn)

listw <- nb2listw(nb, style = "W", zero.policy = TRUE)

return(listw)

}

# ============================================================

# 3. HELPER: GLOBAL MORAN'S I (via spdep)

# ============================================================

run_global_morans <- function(data_sf, var, k, perm = 999) {

x <- as.numeric(data_sf[[var]])

x[is.na(x)] <- 0

listw <- rgeoda_knn_to_listw(data_sf, k)

mt <- moran.mc(x, listw, nsim = perm, zero.policy = TRUE)

data.frame(

variable = var,

k = k,

MoranI = mt$statistic,

p_value = mt$p.value,

interpret = ifelse(mt$p.value < 0.05,

ifelse(mt$statistic > 0, "Clustered", "Dispersed"),

"Random")

)

}

# ============================================================

# 4. HELPER: LOCAL MORAN'S I (via rgeoda — works fine)

# ============================================================

run_local_morans <- function(data_sf, var, k, perm = 999, sig = 0.05) {

w <- knn_weights(data_sf, k = k)

# rgeoda v0.0.1 requires a data.frame, not a vector

x_vec <- as.numeric(data_sf[[var]])

x_vec[is.na(x_vec)] <- 0

x_df <- data.frame(x = x_vec) # <-- KEY FIX

lisa <- local_moran(w, x_df, permutations = perm)

labels <- lisa_labels(lisa)

cl <- lisa_clusters(lisa)

pvals <- lisa_pvalues(lisa)

data_sf[[paste0("LMI_val_", k)]] <- lisa_values(lisa)

data_sf[[paste0("LMI_p_", k)]] <- pvals

data_sf[[paste0("LMI_label_", k)]] <- labels[cl + 1]

data_sf[[paste0("LMI_sig_", k)]] <- ifelse(

pvals <= sig, labels[cl + 1], "Not Significant"

)

return(data_sf)

}

# ============================================================

# 5. HELPER: GETIS-ORD G* (via rgeoda — works fine)

# ============================================================

run_getis_ord <- function(data_sf, var, k, perm = 999, sig = 0.05) {

w <- knn_weights(data_sf, k = k)

x_vec <- as.numeric(data_sf[[var]])

x_vec[is.na(x_vec)] <- 0

x_df <- data.frame(x = x_vec)

lisa <- local_gstar(w, x_df, permutations = perm)

gvals <- lisa_values(lisa)

pvals <- lisa_pvalues(lisa)

# lisa_clusters() returns: 0=Not Sig, 1=High (Hot), 2=Low (Cold)

clusters <- lisa_clusters(lisa)

data_sf[[paste0("Gstar_val_", k)]] <- gvals

data_sf[[paste0("Gstar_p_", k)]] <- pvals

# Use cluster labels directly — this is the correct approach

data_sf[[paste0("Gstar_sig_", k)]] <- case_when(

clusters == 1 ~ "Hot Spot",

clusters == 2 ~ "Cold Spot",

TRUE ~ "Not Significant"

)

return(data_sf)

}

# ============================================================

# 6. GLOBAL MORAN'S I — SENSITIVITY ANALYSIS

# ============================================================

global_results <- list()

for (var in c(inc_vars, "Mean_Inc")) {

for (k in k_values) {

cat("Global Moran's I:", var, "| k =", k, "\n")

res <- tryCatch(

run_global_morans(shp, var, k),

error = function(e) { message(" ERROR: ", e$message); NULL }

)

if (!is.null(res)) global_results[[length(global_results) + 1]] <- res

}

}

global_df <- bind_rows(global_results)

print(global_df)

write.csv(global_df, "Global_MoransI_kNN_Sensitivity.csv", row.names = FALSE)

# --- Plot: Moran's I across k ---

ggplot(global_df %>% filter(variable != "Mean_Inc"),

aes(x = k, y = MoranI, color = variable, group = variable)) +

geom_line(linewidth = 1) + geom_point(size = 2.5) +

geom_hline(yintercept = 0, linetype = "dashed", color = "gray40") +

labs(x = "Number of Neighbors (k)", y = "Moran's I", color = "Year") +

theme_bw() +

theme(axis.text.x = element_text(

size = 12, hjust = 0.5),

axis.text.y = element_text(size = 12),

axis.title.x = element_text(size = 12),

axis.title.y = element_text(size = 12),

legend.text = element_text(size = 12),

legend.title = element_text(size = 12),

panel.grid.major = element_blank(),

panel.grid.minor = element_blank())

ggsave("Global_MoransI_Sensitivity.jpeg", dpi = 600, width = 9, height = 6)

# --- Plot: p-values across k ---

ggplot(global_df %>% filter(variable != "Mean_Inc"),

aes(x = k, y = p_value, color = variable, group = variable)) +

geom_line(linewidth = 1) + geom_point(size = 2.5) +

geom_hline(yintercept = 0.05, linetype = "dashed", color = "red") +

labs(title = "p-value Stability Across k (Global Moran's I)",

x = "k", y = "p-value", color = "Year") +

theme_bw() +

theme(axis.text.x = element_text(

size = 12, hjust = 0.5),

axis.text.y = element_text(size = 12),

axis.title.x = element_text(size = 12),

axis.title.y = element_text(size = 12),

legend.text = element_text(size = 12),

legend.title = element_text(size = 12),

panel.grid.major = element_blank(),

panel.grid.minor = element_blank())

ggsave("Global_MoransI_pvalue_Sensitivity.png", dpi = 600, width = 9, height = 5)

# ============================================================

# 7. LOCAL MORAN'S I — SENSITIVITY + YEARLY MAPS

# ============================================================

lisa_colors <- c(

"High-High" = "#FF0000",

"Low-Low" = "#0000FF",

"High-Low" = "#FF9999",

"Low-High" = "#9999FF",

"Not Significant" = "#D3D3D3"

)

# --- 7a. Sensitivity across k (Mean_Inc) ---

shp_lisa <- shp

for (k in k_values) {

cat("LISA k =", k, "\n")

shp_lisa <- run_local_morans(shp_lisa, "Mean_Inc", k)

}

# Verify all columns created successfully

print(names(shp_lisa)[grepl("LMI_sig", names(shp_lisa))])

# Expected: "LMI_sig_3" "LMI_sig_4" "LMI_sig_5" "LMI_sig_6" "LMI_sig_8" "LMI_sig_10"

# STEP 3: CREATE lisa_sens_plots

# ============================================================

lisa_sens_plots <- lapply(k_values, function(k) {

col <- paste0("LMI_sig_", k)

# Convert to factor with fixed levels so all plots share same legend

shp_lisa[[col]] <- factor(shp_lisa[[col]], levels = names(lisa_colors))

ggplot(shp_lisa) +

geom_sf(aes(fill = .data[[col]]),

color = "white", linewidth = 0.2) +

scale_fill_manual(values = lisa_colors, name = "Cluster",

drop = FALSE, na.value = "#D3D3D3") +

labs(title = paste0("LISA k = ", k)) +

theme_void() +

theme(legend.position = "right",

plot.title = element_text(face = "bold", size = 13),

legend.text = element_text(size = 12),

legend.title = element_text(size = 12))

})

# Confirm it was created

cat("Number of plots created:", length(lisa_sens_plots), "\n") # must be 6

# ============================================================

# STEP 4: SAVE

# ============================================================

ggsave("LISA_Sensitivity_Maps.jpeg",

wrap_plots(lisa_sens_plots, ncol = 3) +

plot_annotation(

title = "Local Moran's I — kNN Sensitivity (Mean Incidence 2017-2024)",

theme = theme(plot.title = element_text(face = "bold", size = 13))

),

dpi = 600, width = 13, height = 8)

cat("✅ LISA sensitivity maps saved!\n")

# --- 7b. Yearly LISA at optimal k ---

shp_yr_lisa <- shp

yr_lisa_plots <- list()

for (yr in 2017:2024) {

var_yr <- inc_vars[grepl(yr, inc_vars)]

if (length(var_yr) == 0) next

shp_yr_lisa <- run_local_morans(shp_yr_lisa, var_yr, k_opt)

yr_lisa_plots[[as.character(yr)]] <- ggplot(shp_yr_lisa) +

geom_sf(aes(fill = .data[[paste0("LMI_sig_", k_opt)]]),

color = "#E0EEEE", linewidth = 0.2) +

scale_fill_manual(values = lisa_colors, name = "Cluster", drop = FALSE) +

labs(title = as.character(yr)) +

theme_void() +

theme(legend.position = "none",

plot.title = element_text(face = "bold", hjust = 0.5, size = 12))

}

# ============================================================

# MODIFY ONLY THE 2017 PLOT TO SHOW LEGEND ON LEFT

# ============================================================

# Extract 2017 plot and add legend back

yr_lisa_plots[["2017"]] <- yr_lisa_plots[["2017"]] +

theme(

legend.position = "left",

legend.title = element_text(face = "bold", size = 12),

legend.text = element_text(size = 11),

legend.key.size = unit(0.3, "cm"),

legend.spacing.y = unit(0.15, "cm")

)

ggsave("LISA_by_Year.jpeg",

wrap_plots(yr_lisa_plots, ncol = 4) +

plot_annotation(

theme = theme(plot.title = element_text(face = "bold", size = 13))

),

dpi = 600, width = 9, height = 6)

# ============================================================

# 8. GETIS-ORD G* — SENSITIVITY + YEARLY MAPS

# ============================================================

gstar_colors <- c(

"Hot Spot" = "#FF0000",

"Cold Spot" = "#0000FF",

"Not Significant" = "#D3D3D3"

)

# --- 8a. Sensitivity across k (Mean_Inc) ---

shp_gstar <- shp

for (k in k_values) {

cat("G* k =", k, "\n")

shp_gstar <- run_getis_ord(shp_gstar, "Mean_Inc", k)

}

gstar_sens_plots <- lapply(k_values, function(k) {

ggplot(shp_gstar) +

geom_sf(aes(fill = .data[[paste0("Gstar_sig_", k)]]),

color = "white", linewidth = 0.2) +

scale_fill_manual(values = gstar_colors, name = "Spot", drop = FALSE) +

labs(title = paste0("G* k = ", k)) +

theme_void() +

theme(legend.position = "right",

plot.title = element_text(face = "bold", size = 13),

legend.text = element_text(size = 12),

legend.title = element_text(size = 12))

})

ggsave("Gstar_Sensitivity_Maps.png",

wrap_plots(gstar_sens_plots, ncol = 3) +

plot_annotation(

title = "Getis-Ord G* — kNN Sensitivity (Mean Incidence 2017-2024)",

theme = theme(plot.title = element_text(face = "bold", size = 13))

),

dpi = 600, width = 13, height = 8)

# --- 8b. Yearly G* at optimal k ---

shp_yr_gstar <- shp

yr_gstar_plots <- list()

for (yr in 2017:2024) {

var_yr <- inc_vars[grepl(yr, inc_vars)]

if (length(var_yr) == 0) next

shp_yr_gstar <- run_getis_ord(shp_yr_gstar, var_yr, k_opt)

yr_gstar_plots[[as.character(yr)]] <- ggplot(shp_yr_gstar) +

geom_sf(aes(fill = .data[[paste0("Gstar_sig_", k_opt)]]),

color = "#E0EEEE", linewidth = 0.2) +

scale_fill_manual(values = gstar_colors, name = "Spot", drop = FALSE) +

labs(title = as.character(yr)) +

theme_void() +

theme(legend.position = "none",

plot.title = element_text(face = "bold", hjust = 0.5, size = 12))

}

#modify 2017 plot to have legend

yr_gstar_plots[["2017"]] <- yr_gstar_plots[["2017"]] +

theme(

legend.position = "left",

legend.title = element_text(face = "bold", size = 12),

legend.text = element_text(size = 11),

legend.key.size = unit(0.3, "cm"),

legend.spacing.y = unit(0.15, "cm")

)

ggsave("Gstar_by_Year.jpeg",

wrap_plots(yr_gstar_plots, ncol = 4) +

plot_annotation(

theme = theme(plot.title = element_text(face = "bold", size = 13))

),

dpi = 600, width = 9, height = 6)

# ============================================================

# 9. SENSITIVITY AGREEMENT MAP (LISA)

# ============================================================

lisa_sig_cols <- paste0("LMI_sig_", k_values)

agree_df <- st_drop_geometry(shp_lisa)[, lisa_sig_cols]

shp_lisa$dominant_cluster <- apply(agree_df, 1,

function(x) names(which.max(table(x))))

shp_lisa$k_agreement <- apply(agree_df, 1,

function(x) max(table(x)) / length(k_values)) # proportion of k agreeing

ggplot(shp_lisa) +

geom_sf(aes(fill = dominant_cluster, alpha = k_agreement),

color = "white", linewidth = 0.2) +

scale_fill_manual(values = lisa_colors, name = "Cluster Type") +

scale_alpha_continuous(range = c(0.25, 1.0),

name = "Proportion k\nin agreement") +

labs(title = "Most Consistent LISA Cluster Across kNN Sensitivity",

subtitle = "Opacity reflects agreement across k = 3,4,5,6,8,10") +

theme_void()

ggsave("LISA_Agreement_Map.jpeg", dpi = 600, width = 10, height = 8)

# ============================================================

# 10. EXPORT RESULTS

# ============================================================

st_write(shp_lisa, "LISA_Results.shp", delete_dsn = TRUE)

st_write(shp_gstar, "Gstar_Results.shp", delete_dsn = TRUE)

st_write(shp_yr_lisa, "LISA_by_Year.shp", delete_dsn = TRUE)

st_write(shp_yr_gstar, "Gstar_by_Year.shp", delete_dsn = TRUE)

cat("\n✅ Analysis complete! All outputs saved.\n")

## Spatiotemporal Cluster Analysis

# ============================================================

# SaTScan v10.1.3 (Windows) - Retrospective Space-Time Permutation (STP)

# Monthly data | High rates | Monte Carlo reps: 999

# Study period: 2016/01/01 to 2024/12/31

# final_data.csv: Location_ID, Year, Month, Cases, latitude, longitude

# Output: C:/SaTScan_R_runs/kenny_run_auto_fromR

# ============================================================

# -----------------------------

# 0) PACKAGES

# -----------------------------

pkgs <- c("dplyr", "readr")

to_install <- pkgs[!pkgs %in% installed.packages()[, "Package"]]

if (length(to_install) > 0) install.packages(to_install)

library(dplyr)

library(readr)

library(rsatscan)

# -----------------------------

# 1) PATH SETTINGS

# -----------------------------

in_csv <- "P:/01 Work/1_RESEARCH/Dengue_Kenny_Philippins/R_SaTScan_Kenny/final_data.csv"

run_dir <- "C:/SaTScan_R_runs/kenny_run_auto_fromR"

dir.create(run_dir, recursive = TRUE, showWarnings = FALSE)

satscan_exe <- "C:/Program Files/SaTScan/SaTScanBatch64.exe"

stopifnot(file.exists(in_csv))

stopifnot(file.exists(satscan_exe))

# -----------------------------

# 2) READ + VALIDATE

# -----------------------------

dat <- read_csv(in_csv, show_col_types = FALSE)

need_cols <- c("Location_ID", "Year", "Month", "Cases", "latitude", "longitude")

miss <- setdiff(need_cols, names(dat))

if (length(miss) > 0) stop("Missing columns: ", paste(miss, collapse = ", "))

dat <- dat %>%

mutate(

Location_ID = as.integer(Location_ID),

Year = as.integer(Year),

Month = as.integer(Month),

Cases = as.numeric(Cases),

latitude = as.numeric(latitude),

longitude = as.numeric(longitude)

) %>%

filter(Year >= 2016 & Year <= 2024)

if (anyNA(dat$Location_ID) || anyNA(dat$Year) || anyNA(dat$Month) || anyNA(dat$Cases)) {

stop("NA found in Location_ID / Year / Month / Case")

}

if (any(dat$Month < 1 | dat$Month > 12)) stop("Month must be between 1 and 12")

# -----------------------------

# 3) BUILD CASE FILE (.cas)

# *** IMPORTANT: format must be: LocationID Cases Date ***

# Date must match PrecisionCaseTimes=2 (Month) -> use YYYY/MM/01

# -----------------------------

cas_df <- dat %>%

group_by(Location_ID, Year, Month) %>%

summarise(Cases = sum(Cases, na.rm = TRUE), .groups = "drop") %>%

mutate(

Cases = as.integer(round(Cases)),

Date = sprintf("%04d/%02d/01", Year, Month)

) %>%

select(Location_ID, Cases, Date) %>%

arrange(Location_ID, Date)

case_file <- file.path(run_dir, "kenny.cas")

write.table(cas_df, case_file, row.names = FALSE, col.names = FALSE,

quote = FALSE, sep = " ")

cat("Case head:\n")

print(readLines(case_file, n = 5))

# should look like: 1 13 2017/01/01

# -----------------------------

# 4) BUILD GEO FILE (.geo)

# format: LocationID lat long

# -----------------------------

geo_df <- dat %>%

filter(!is.na(latitude), !is.na(longitude)) %>%

group_by(Location_ID) %>%

summarise(

latitude = latitude[1],

longitude = longitude[1],

n_coord = n_distinct(paste(latitude, longitude)),

.groups = "drop"

)

multi <- geo_df %>% filter(n_coord > 1)

if (nrow(multi) > 0) {

warning("Some Location_ID has multiple coordinates -> Use the first row, e.g.: ",

paste(head(multi$Location_ID, 10), collapse = ", "))

}

geo_out <- geo_df %>% select(Location_ID, latitude, longitude) %>% arrange(Location_ID)

geo_file <- file.path(run_dir, "kenny.geo")

write.table(geo_out, geo_file, row.names = FALSE, col.names = FALSE,

quote = FALSE, sep = " ")

cat("Geo head:\n")

print(readLines(geo_file, n = 5))

# -----------------------------

# 5) RUN SaTScanBatch (command-line)

# +++ ADD: KML + SHP outputs +++

# -----------------------------

case_full <- normalizePath(case_file, winslash = "/", mustWork = TRUE)

geo_full <- normalizePath(geo_file, winslash = "/", mustWork = TRUE)

exe_full <- normalizePath(satscan_exe, winslash = "/", mustWork = TRUE)

results_prefix <- normalizePath(file.path(run_dir, "kenny_results"),

winslash = "/", mustWork = FALSE)

log_file <- file.path(run_dir, "kenny_run_log.txt")

args <- c(

"--CaseFile", case_full,

"--CoordinatesFile", geo_full,

"--CoordinatesType", "1",

"--ResultsFile", results_prefix,

"--AnalysisType", "3", # Retrospective Space-Time

"--ModelType", "2", # Space-Time Permutation

"--ScanAreas", "1", # High rates

"--PrecisionCaseTimes", "2", # Month

"--StartDate", "2016/01/01",

"--EndDate", "2024/12/31",

"--TimeAggregationUnits", "2", # Month

"--TimeAggregationLength", "1",

"--SpatialWindowShapeType", "0",

"--MaxSpatialSizeInPopulationAtRisk", "50",

"--MinimumTemporalClusterSize", "1",

"--MaxTemporalSizeInterpretation", "0",

"--MaxTemporalSize", "50",

"--MinimumCasesInHighRateClusters", "2",

"--MonteCarloReps", "999",

# -----------------------------

# OUTPUT: KML + SHP

# -----------------------------

"--OutputGoogleEarthKML", "y", # create KML/KMZ outputs

"--CompressKMLtoKMZ", "y", # create KMZ (compressed)

"--OutputShapefiles", "y", # create shapefiles for GIS (QGIS/ArcGIS)

"--PrintAsciiColumnHeaders", "n"

)

old <- getwd()

on.exit(setwd(old), add = TRUE)

setwd(run_dir)

out <- system2(exe_full, args = args, stdout = TRUE, stderr = TRUE)

writeLines(out, log_file)

cat("\n=== Tail log ===\n")

cat(paste(tail(out, 80), collapse = "\n"), "\n")

# -----------------------------

# 6) CHECK OUTPUT

# -----------------------------

cat("\n=== Files in run_dir ===\n")

files <- list.files(run_dir, full.names = TRUE)

print(basename(files))

cat("\n=== Key expected outputs ===\n")

expected <- c(

paste0(results_prefix, ".txt"),

paste0(results_prefix, ".kmz"),

paste0(results_prefix, ".kml"),

paste0(results_prefix, ".shp"),

paste0(results_prefix, ".dbf"),

paste0(results_prefix, ".shx")

)

print(data.frame(file = basename(expected), exists = file.exists(expected)))

main_report <- paste0(results_prefix, ".txt")

cat("\n=== Done ===\n")

cat("Log file: ", log_file, "\n")

cat("Open KMZ/KML in Google Earth, and SHP in QGIS/ArcGIS.\n")
